# *paRDal*: bioinformatics framework for a parallel RNA and DNA NGS analysis in hereditary cancer diagnostics

**DOI:** 10.3389/fbinf.2026.1944540

**Published:** 2026-10-20

**Authors:** Katerina Matejkova, Petr Nehasil, Petra Kleiblova, Tatana Ptackova, Marta Cerna, Pavel Just, Ondrej Vit, Petra Zemankova, Mária Giertlova, Marketa Janatova, Jana Soukupova, Ina Skaara Brorson, Zdenek Kleibl

**Affiliations:** 1 Institute of Medical Biochemistry and Laboratory Diagnostics, First Faculty of Medicine, Charles University and General University Hospital in Prague, Prague, Czechia; 2 Department of Genetics and Microbiology, Faculty of Science, Charles University, Prague, Czechia; 3 Department of Paediatrics and Inherited Metabolic Disorders, First Faculty of Medicine, Charles University and General University Hospital in Prague, Prague, Czechia; 4 Institute of Pathological Physiology, First Faculty of Medicine, Charles University, Prague, Czechia; 5 Institute of Biology and Medical Genetics, First Faculty of Medicine, Charles University and General University Hospital in Prague, Prague, Czechia; 6 BIOCEV Biotechnology and Biomedicine Center, First Faculty of Medicine, Charles University, Vestec, Czechia; 7 Department of Clinical Neurosciences, Faculty of Medicine, University of PJ Safarik in Kosice, Kosice, Slovakia; 8 Department of Medical Genetics, Children’s Faculty Hospital and Slovak Health University in Banska Bystrica, Banska Bystrica, Slovakia; 9 Department of Medical Genetics, Oslo University Hospital, Oslo, Norway; 10 Department of Medical Genetics, Institute of Clinical Medicine, Faculty of Medicine, University of Oslo, Oslo, Norway

**Keywords:** ACMG criteria, multiomics, parallel DNA/RNA analysis, splicing analysis, targeted DNA/RNA NGS, variant classification

## Abstract

Germline next-generation sequencing (NGS) is the gold standard for hereditary cancer diagnostics. However, clinical utility is limited by variants of uncertain significance (VUS), which may disrupt mRNA processing; consequences invisible to DNA NGS alone but identifiable by parallel RNA NGS. Here, we present *paRDal* (parallel RNA/DNA analytical workflow), a containerized, modular framework that integrates matched germline DNA and RNA NGS data into a single diagnostic pipeline. *paRDal* implements allele-aware two-pass alignment, automated splicing and expression outlier detection, allele-specific expression analysis, and comprehensive variant annotation. A bidirectionally reactive filtering engine enables both DNA-centric (variant-to-function) and RNA-centric (function-to-variant) workflows. An R/Shiny application consolidates multilayered evidence per patient and across cohorts, linking *in silico* predictions with observed transcriptomic consequences without requiring specialized bioinformatic expertise. As demonstrated in 204 individuals undergoing hereditary cancer genetic testing, *paRDal* facilitates the standardized application of RNA-based ACMG/AMP criteria [PVS1(RNA), PS3, PP3, BP7(RNA)] supporting VUS resolution.

## 1 Background

Next-generation sequencing (NGS) of germline DNA has become the gold standard for the analysis of hereditary cancer predisposition, currently the most common application of Mendelian disease diagnostics in human clinical genetics, increasing diagnostic yield while reducing turnaround time and costs (Kuzbari et al., 2023; Ewans et al., 2022; Smedley et al., 2021; Wojcik et al., 2024; Huang et al., 2018). Beyond the identification of causal germline pathogenic variants (gPV) in cancer predisposition genes, which enables the application of tailored cancer prevention strategies for high-risk carriers, NGS reveals many variants of uncertain significance (VUS). These genetic changes, with unknown biological effects and clinical implications, hamper the diagnostic utility of NGS and may lead to inadequate disease surveillance (Burke et al., 2022). VUS account for approximately 40% of NGS-identified variants (Federici and Soddu, 2020; Chen et al., 2023) and are found in up to 30% of patients (Federici and Soddu, 2020; Ha et al., 2020). Their resolution has accelerated in recent years, largely driven by machine-learning approaches leveraging large public variant datasets to provide more quantitative assessment (Kobayashi et al., 2024).

RNA NGS offers a complementary layer of evidence for resolving VUS, particularly those affecting mRNA processing. By capturing transcript-level consequences, RNA NGS can reveal the impact of variants on pre-mRNA splicing, nonsense-mediated decay (NMD), and gene expression, none of which are fully assessable by DNA NGS (Horton et al., 2024; Rhine et al., 2018; Li et al., 2016; Buzby et al., 2017; Evans et al., 2018; Perrin-Vidoz et al., 2002). About 13% of all gPVs disrupt canonical splice sites (±1/2 positions of donor or acceptor sites), leading to aberrant splicing. However, non-canonical splicing variants, such as those at exonic and especially deep-intronic positions, are likely substantially underreported (Horton et al., 2024; Rhine et al., 2018; Dragoš et al., 2022; Truty et al., 2021), as they often evade detection and prioritization in panel-based or whole-exome sequencing (WES) approaches. Incorporating RNA NGS into clinical workflows has shown considerable potential for reclassifying canonical and non-canonical splice variants alike, yielding mechanistic evidence for pathogenicity that supports more accurate diagnosis and clinical decision-making (Chen et al., 2023; Yépez et al., 2022). Furthermore, RNA NGS can identify aberrant allele-specific expression and gene expression outliers.

Several studies have applied RNA NGS to improve variant interpretation in clinical settings, particularly for evaluating aberrant splicing, expression outliers, and allele-specific expression, as well as for variant calling and sample authentication (Truty et al., 2021; Yépez et al., 2022; Jaramillo Oquendo et al., 2024; Agiannitopoulos et al., 2021; Frésard et al., 2019; De Cock et al., 2025; Sha et al., 2025; Luo et al., 2025; Yépez et al., 2021; Kamps-Hughes et al., 2023; Kleiblová et al., 2024). Importantly, the American College of Medical Genetics and Genomics (ACMG) and the Association for Molecular Pathology (AMP) have acknowledged the role of RNA NGS and splicing prediction tools as evidence for variant reclassification. Under the ACMG/AMP framework, RNA NGS can support the PVS1(RNA) criterion, yet its implementation remains challenging, partly due to the lack of standardized frameworks and practical guidance (Walker et al., 2023; Brnich et al., 2019).

RNA NGS-based workflows typically rely on a combination of tools for splice junction or expression outlier detection, and allele-specific expression analysis (Yépez et al., 2022; Yépez et al., 2021). However, no standardized or widely adopted framework currently exists that systematically integrates DNA NGS with RNA NGS into a variant interpretation workflow, particularly in the context of hereditary cancer diagnostics. While some frameworks address isolated aspects, none covers the full optimized workflow: from raw data processing and read mapping through variant calling to comprehensive variant annotation and functional RNA analysis. This fragmentation drives variability in analytical approaches and inconsistent application of RNA-based evidence across clinical genomics laboratories. Additionally, the implementation of functional evidence in clinical settings is frequently constrained by a critical technical bottleneck: fragmented workflows generate complex, multilayered data that remain inaccessible to geneticists and clinicians lacking specialized bioinformatic expertise.

Here we present *paRDal* (parallel RNA/DNA analytical workflow), a containerized (Docker) modular framework designed for the systematic integration of matched DNA and RNA NGS, empowering clinicians and researchers to efficiently examine patient genomic data. *paRDal* integrates established tools (BWA-MEM, STAR, GATK, VEP, FRASER, OUTRIDER, MBASED) rather than replacing them; its contribution lies in end-to-end integration, standardized implementation, and an interactive clinical interface. The workflow comprises optimized pre-processing with allele-aware two-pass alignment, followed by variant calling, expression and splicing outlier detection, allele-specific expression analysis, and sample authentication, all consolidated into an R/Shiny application that makes the multilayered output directly interpretable.

## 2 Methods

### 2.1 Dataset and NGS

Paired DNA and RNA samples were collected from 204 individuals of Czech and Slovak descent who underwent germline genetic testing for hereditary cancer predisposition at the General University Hospital in Prague (GUHP). The study was approved by the Ethics Committee of the GUHP (ethics approval number 22/22). All participants provided informed consent, and the study was conducted in accordance with the Declaration of Helsinki. DNA and RNA were isolated from peripheral blood leukocytes or cultured lymphocytes as described previously (Kleiblová et al., 2024; Černá et al., 2026; Černá et al., 2025). DNA and RNA panel NGS libraries were prepared using the custom CZECANCA panel (CZEch CAncer paNel for Clinical Application) as described previously (Kleiblová et al., 2024; Soukupova et al., 2018), and sequenced using Illumina paired-end sequencing on NextSeq 2000. Fifty matched genomes from 1000 Genomes (Auton et al., 2015) and transcriptomes from Geuvadis project (Lappalainen et al., 2013) served as external controls and were analyzed in a separate run.

### 2.2 General workflow

Raw NGS data were processed using a fully containerized modular workflow (Figure 1) implemented inside Docker, ensuring complete reproducibility of software versions, dependencies, and reference files. All steps, tools, folder structures, and scripts used in this workflow are defined in our GitHub repository (see Section 2.11).

**FIGURE 1:**
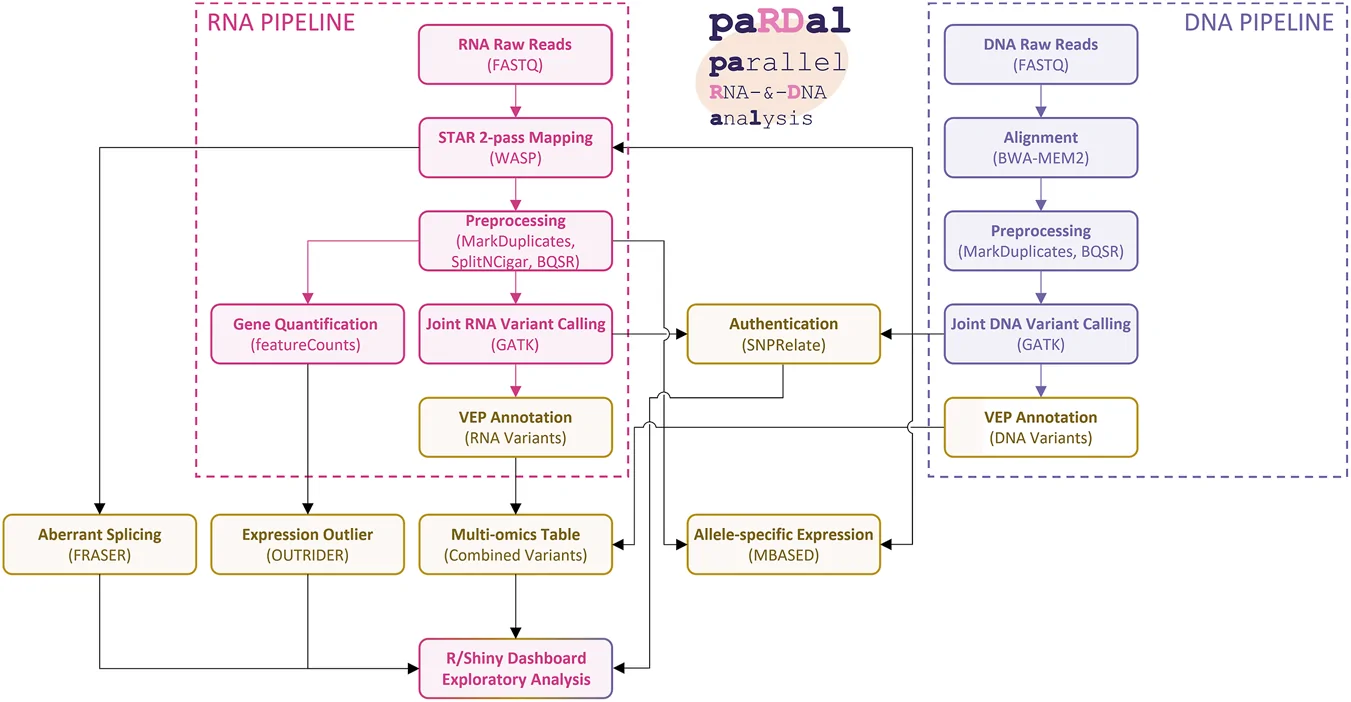
*paRDal* workflow. The flowchart describes basic functionalities of the framework and software tools used.

All mapping steps, variant calling and annotation steps used the GRCh38 primary assembly (Homo_sapiens.GRCh38.dna.primary.assembly.fa, release 114) and Ensembl GRCh38 GTF annotation file (release 114) (Dyer et al., 2025). All databases used for annotation, as well as the tools and their versions, are listed in Supplementary Tables S1, S2, respectively.

### 2.3 DNA NGS data preprocessing and variant calling

Raw DNA NGS paired-end reads were processed according to GATK Best Practices workflow (Van der Auwera et al., 2013). Raw FASTQ files were aligned to the reference genome using BWA-MEM2 (v2.0) (Vasimuddin et al., 2019). Sorting, duplicate marking, and read groups assignment were performed with Picard tools (v1.129) (Broad Institute, 2019). Base Quality Score Recalibration (BQSR) was applied using GATK (v4.3.0.0) (Van der Auwera et al., 2013) BaseRecalibrator, employing sets of known, high-confidence variants (Mills and 1000G gold standard indels; 1000G phase 1 SNPs) (Van der Auwera et al., 2013).

Variant calling was conducted per-sample using GATK (v4.3.0.0) HaplotypeCaller in GVCF mode. Cohort-wide joint genotyping was subsequently performed using GATK (v4.3.0.0) GenotypeGVCFs. Multiallelic variants were decomposed, and indels were left-aligned and normalized using BCFtools (v1.9) (Danecek and McCarthy, 2017) and vt (v0.57721) (Tan et al., 2015). Standard cohort-level hard filtering was applied to retain high-quality variants (DP ≥ 5, FS < 45, QD > 1, SOR < 4, MQRankSum ≥ −13.5).

Single-nucleotide variants (SNVs) were extracted to support RNA NGS allele-aware alignment using the WASP algorithm and allele-specific expression (ASE) analysis (van de Geijn et al., 2015). Annotation was carried out using Ensembl Variant Effect Predictor (VEP) (McLaren et al., 2016) with multiple plugins, as described in Supplementary Table S1.

### 2.4 RNA NGS data preprocessing and variant calling

RNA NGS paired-end reads were aligned using STAR (v2.7.11b) utilizing a rigorous two-pass mapping mode that supports the discovery of novel splice junctions (Dobin et al., 2013). First pass mapping required an 8-bp overhang for *de novo* junctions, whereas only a 3-bp overhang was needed for annotated junctions. Export to sample-level databases was strictly gated by a 12-bp bilateral overhang; minimum coverage of three reads was required for non-canonical junctions, whereas coverage of one read sufficed for canonical junctions. High-confidence novel junctions from the whole cohort were subsequently utilized to generate updated genome indices for the second pass. The second pass required a minimum depth of three reads across all canonical and non-canonical motifs for the export to the final splice-junctions file. To suppress reference-mapping bias, a critical requirement for accurate ASE analysis, the second alignment pass was executed with the WASP algorithm enabled. During this step, patient-matched variants derived from the DNA variant calling pipeline were supplied as known variant loci.

RNA BAMs were processed following a workflow analogous to the DNA pipeline, with necessary adaptations for transcriptome data. PCR duplicates were removed using Picard tools (v1.129) (Broad Institute, 2019); however, the pipeline includes an option to bypass this step for highly expressed datasets. To ensure compatibility with downstream variant calling, spliced reads were processed using SplitNCigarReads (GATK, v4.3.0.0) (Van der Auwera et al., 2013). Variant calling was performed using HaplotypeCaller with soft-clipped bases omitted to minimize false positive calls near splice junctions. Standard cohort-level hard filtering was applied to retain high-quality variants using the same parameters as the DNA workflow (DP ≥ 5, FS < 45, QD > 1, SOR < 4, MQRankSum ≥ −13.5).

### 2.5 Variant annotation

Normalized DNA/RNA variants were comprehensively annotated using Ensemble VEP (offline cache mode, GRCh38, release 114) (McLaren et al., 2016). A predefined list of clinically significant transcription variants in cancer predisposition genes was used for internal samples. MANE Select transcripts (Morales et al., 2022) were used for the transcriptomes from the Geuvadis project.

To empower the diagnostic evaluation of multiomics outliers and aberrant events, the annotation framework was augmented with a specialized set of plugins categorized by biological mechanism (Supplementary Table S1).

By integrating a multilayered splicing prediction framework, we provided a mechanistic background for aberrant splicing outliers detected by FRASER (Mertes et al., 2021). The SpliceAI and SpliceVault plugins were utilized to predict cryptic donor/acceptor activation, intron retention, and exon skipping events (Jaganathan et al., 2019; Dawes et al., 2023). Standard motifs were annotated using the SpliceRegion plugin.

To facilitate interpretation of protein dysfunction in the absence of transcriptomic outliers, deleteriousness-prediction tools were incorporated, including AlphaMissense (Cheng et al., 2023), BayesDel (Feng, 2017), CADD (Kircher et al., 2014), REVEL (Ioannidis et al., 2016), and SIFT (Ng and Henikoff, 2001) plugins. To further elucidate the variants’ impact on protein structure and function, the DOMAINS and ProteinLengthChange plugins were included to assess domain disruption and changes in protein length. MaveDB (Esposito et al., 2019) provides functional experimental variant effects.

Variants were placed in context using global population frequencies and clinical databases, using gnomAD (v4.0) and the ClinVar database (obtained 11/2025), to ensure clinical relevance. Furthermore, the annotation logic prioritizes clinical transcripts, integrating RefSeq and MANE Select identifiers to ensure that HGVSc and HGVSp (den Dunnen et al., 2016) notations are consistent with established ACMG/AMP variant classification standards.

Annotated variants from both DNA and RNA molecular layers were merged into a final comprehensive table. Variant detection across DNA, RNA, or both was encoded using binary indicators, alongside cohort-level carrier counts for each category. Concordance between the two layers was evaluated using the RNA-to-DNA Variant Allele Frequency (VAF) ratio; variants with a ratio between 0.8 and 1.2 were classified as expected. The pipeline also explicitly flags intronic variants to highlight their expected absence or unexpected presence in the transcriptome. Finally, within the interactive interface, high-confidence variants defined as having a genotype quality (GQ) of 99 and a read depth (DP) ≥ 10 in either or both layers are prioritized for downstream analysis.

### 2.6 Gene expression quantification and expression outlier detection with OUTRIDER

Gene-level read counts were quantified from the final RNA BAM files using featureCounts from the Subread package (v2.0.2), counting paired reads spanning exons. Reads were not normalized, leaving normalization to the user.

Expression outliers were detected using the OUTRIDER package in R (Brechtmann et al., 2018). Genes with low expression were strictly filtered out (FPKM < 10). OUTRIDER’s denoising autoencoder was applied to correct for unobserved confounders. Significant expression outliers were defined as events exhibiting an adjusted p-value <0.05.

### 2.7 Aberrant splicing detection and splicing outlier identification using FRASER

Splicing outliers were extracted from RNA NGS BAMs using the FRASER R-package (Mertes et al., 2021), followed by the calculation of the Percent Spliced In ( Ψ ). Significant aberrant splicing events were strictly defined using an adjusted p-value <0.05, an absolute ΔΨ > 0.1, and a minimum junction coverage of five reads. FRASER was used to generate Sashimi plots for the aberrant splice junctions.

### 2.8 Allele-specific expression (ASE)

ASE profiling was restricted to exonic heterozygous, biallelic SNPs. Allelic counts were quantified directly from the RNA BAM files using GATK ASEReadCounter with a minimum required depth of 10 reads for heterozygous sites, with at least five reads for both the reference and alternative alleles. Statistical assessment of allelic imbalance was performed on a gene-level using the MBASED R-package (1-sample analysis mode, unphased, 10^5^ simulations). Significant ASE events were defined by an adjusted (Holm correction) p-value <0.05, a heterogeneity p-value ≥0.05, and a major allele frequency (mAF) < 0.4 or >0.6. As p-values are permutation-derived, they are bounded below by the number of simulations and are reported as p < 1 × 10^−5^ where no simulation exceeded the observed statistic.

### 2.9 Sample authentication

The sample authentication was performed via SNPRelate R-package (Zheng et al., 2012) using Identity-by-Descent measures in order to detect sample swaps or errors in library preparation. Biallelic SNPs were LD-pruned (MAF >0.005; missing rate <0.05), and the KING method was used to confirm autologous pairings. To define the threshold for identifying identical samples, we examined the distribution of kinship coefficients within each pair of matched DNA/RNA samples, and the final cut-off was set to the higher of the two kinship values surrounding the maximum delta if it exceeded 0.4.

### 2.10 Multiomics data integration

The final diagnostic table was constructed using the R/*tidyverse*-based framework. Variant annotation was joined with unannotated DNA and RNA VCFs. DNA and RNA sequencing metrics, including depth and variant allele frequency, were aligned side-by-side to allow immediate distinction between technical artifacts and biologically relevant phenomena such as nonsense-mediated decay (NMD), monoallelic expression, or tissue-specific expression (column details in Supplementary Table S3).

The R/Shiny application was developed with an AI-assisted coding (Google, 2026) approach. However, the multilayer integration logic and data architecture were fully defined by the authors. The application underwent rigorous testing and debugging. Results were thoroughly manually checked for biological sanity, using internal clinical data as positive controls, mimicking real-life manual data curation and evaluation to verify the correctness of filtering and join engines.

Splicing-related ACMG/AMP criteria, including PVS1(RNA), PS3 and BP7_STR(RNA), were assigned according to the ClinGen SVI splicing recommendations (Walker et al., 2023).

### 2.11 Software implementation and containerization

The *paRDal* framework is implemented as a fully containerized solution using Docker (20.10.21) to ensure both fast setup and reproducibility across diverse institutional environments. All required bioinformatics tools, reference genomes (GRCh38), and external databases are included within the Docker image (sha256:7080951ef8683f70f16df97614065a36e6a09b45fdd4119fce116f3662ac1889) that has 9.5 GB and databases require about 950 GB of free space. The setup process and directory structure are described on https://doi.org/10.5281/zenodo.19055361 (https://github.com/LOG-CZECANCA/pardal). The application setup process is described at https://doi.org/10.5281/zenodo.19055389 (https://github.com/LOG-CZECANCA/pardal-app).

### 2.12 Code availability

The underlying code for the *paRDal* workflow and R/Shiny application can be found at https://doi.org/10.5281/zenodo.19055361 and https://doi.org/10.5281/zenodo.19055389.

## 3 Results

### 3.1 Performance and software testing on a cohort of 204 individuals

To demonstrate the scalability of *paRDal*, we benchmarked its performance across two distinct hardware architectures using both targeted clinical panels (CZECANCA) and whole genome/transcriptome pairs.

On a high-end server (AMD EPYC 9654P 96-core, 768 GB RAM), the clinical cohort of 204 individuals sequenced on the CZECANCA panel was processed in 9 h, representing a peak aggregate throughput of 22.7 samples per hour. Processing 50 whole genome/transcriptome pairs from the 1,000 Genomes (Auton et al., 2015) and Geuvadis (Lappalainen et al., 2013) projects required 106.5 h, yielding a throughput of 0.47 samples per hour.

Pipeline performance was also tested on a standard workstation (2x Intel Xeon E5-2690 v2, 128 GB RAM), where 30 individuals analyzed by the CZECANCA panel were processed in 11 h (2.7 samples per hour), demonstrating that *paRDal* performs robustly even on older infrastructure. Mean compressed file sizes were 250 MB for DNA and 215 MB for RNA (targeted panels).

In unfiltered data, we identified 98,325 unique variants across 204 individuals (839,163 variant-individual pairs). To identify candidate causal variants and create a prioritized list for the R/Shiny application, we filtered the original dataset to retain those with a minor allele frequency <1%, a valid feature (e.g., transcript or regulatory feature), and non-benign ClinVar annotations. High-confidence variant calls were defined as those with a minimum read depth ≥10 and the highest genotype quality score of 99 (GQ = 99) in either the matched DNA or RNA sequencing data. We required this quality threshold in only one modality rather than both to account for capture biases: deep-intronic variants missed by targeted DNA NGS (e.g., exome sequencing) may only be confidently detected in RNA (e.g., via aberrant splicing), whereas variants in lowly expressed transcripts or skipped exons may only be reliably called in DNA. The final dataset comprised 5,497 unique candidate variants across 204 individuals.

### 3.2 Integration of DNA and RNA evidence

To facilitate rapid identification and prioritization of disease-causing germline variants, with an emphasis on splicing and non-coding variants, we developed an interactive, R/Shiny-based application that bridges genomic data (DNA and RNA variants) with functional transcriptomic evidence (RNA NGS). The application is structured around two primary analytical modules: a cohort-wide global “All Variants” view and a patient-centric “Patient View” (Figure 2).

**FIGURE 2:**
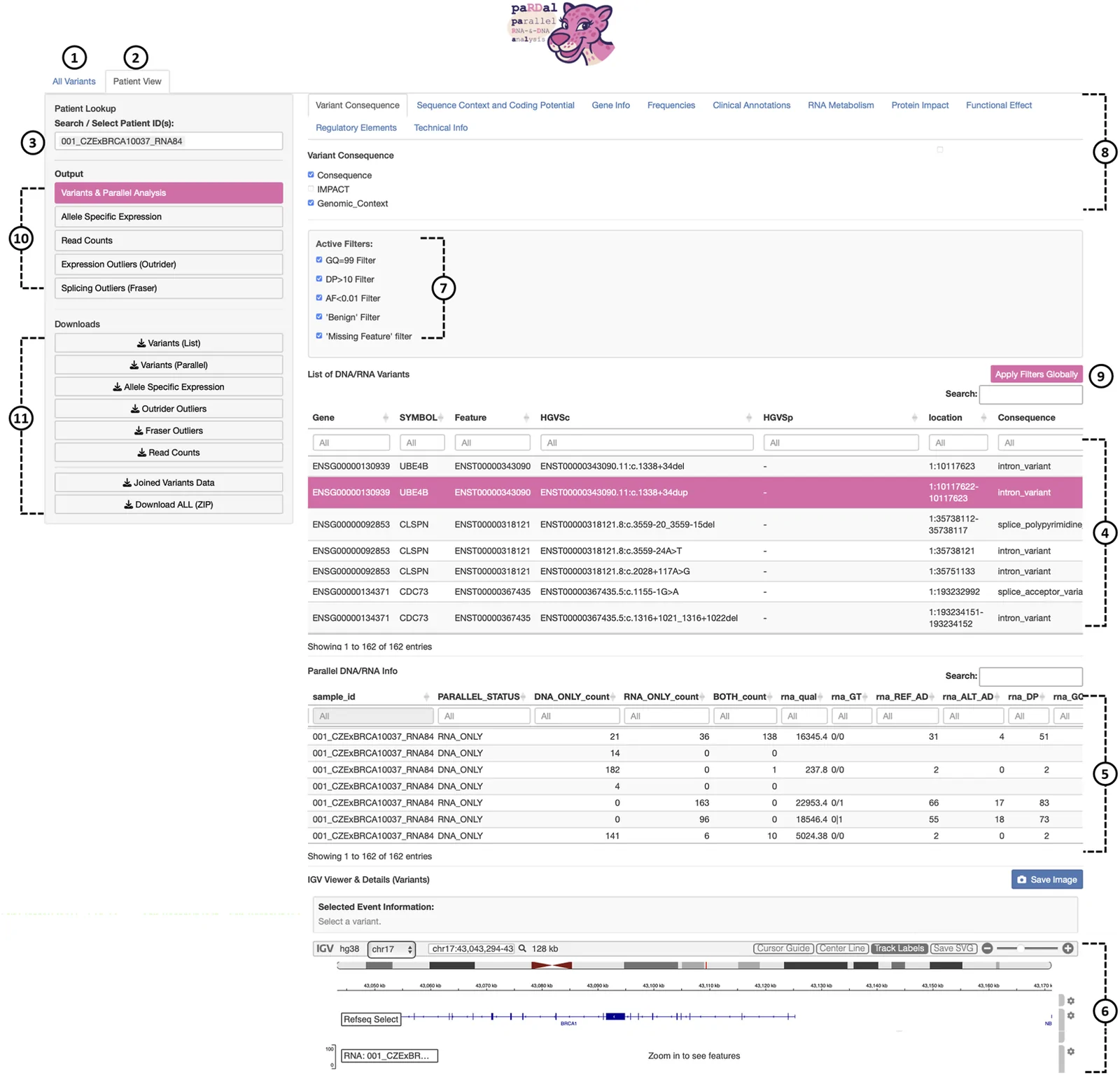
The *paRDal* application layout. Users can toggle between All Variants view (1) displaying all variants across the analyzed cohort (e.g., sequencing run) or Patient View (2), showing variants identified in a given individual (3). List of DNA/RNA Variants (4) includes all variants detected by parallel DNA and RNA NGS, Parallel DNA/RNA Info (5) displays their quality metrics. Clicking a selected variant (not shown) loads it into parallel DNA/RNA tracks in IGV (6). Variants can be prefiltered using switchable filters (7) and annotated using VEP plugins organized into thematically relevant groups (8). The Apply Filters Globally (9) propagates a selected feature (e.g., variant or gene) across all functional output layers (10), which can be downloaded in csv format (11).

DNA/RNA variants are annotated with their functional and structural consequences, protein deleteriousness scores, splicing predictions, population frequencies, and clinical relevance. Annotated variants are displayed in the List of DNA/RNA Variants, with selectable columns or column groups tailored to the specific research or diagnostic question (Supplementary Table S1).

#### 3.2.1 Integrated quality control via side-by-side DNA and RNA variant metrics

To facilitate rigorous technical validation and biological interpretation of identified variants, the application features a parallel analysis module (Parallel DNA/RNA Info) that resolves variant-level data at the individual patient level. While cohort-level summaries are useful for exploratory data analysis, confidently interpreting a causal variant requires assessing its sequencing quality and expression status at the individual level. (Figure 3)

**FIGURE 3:**
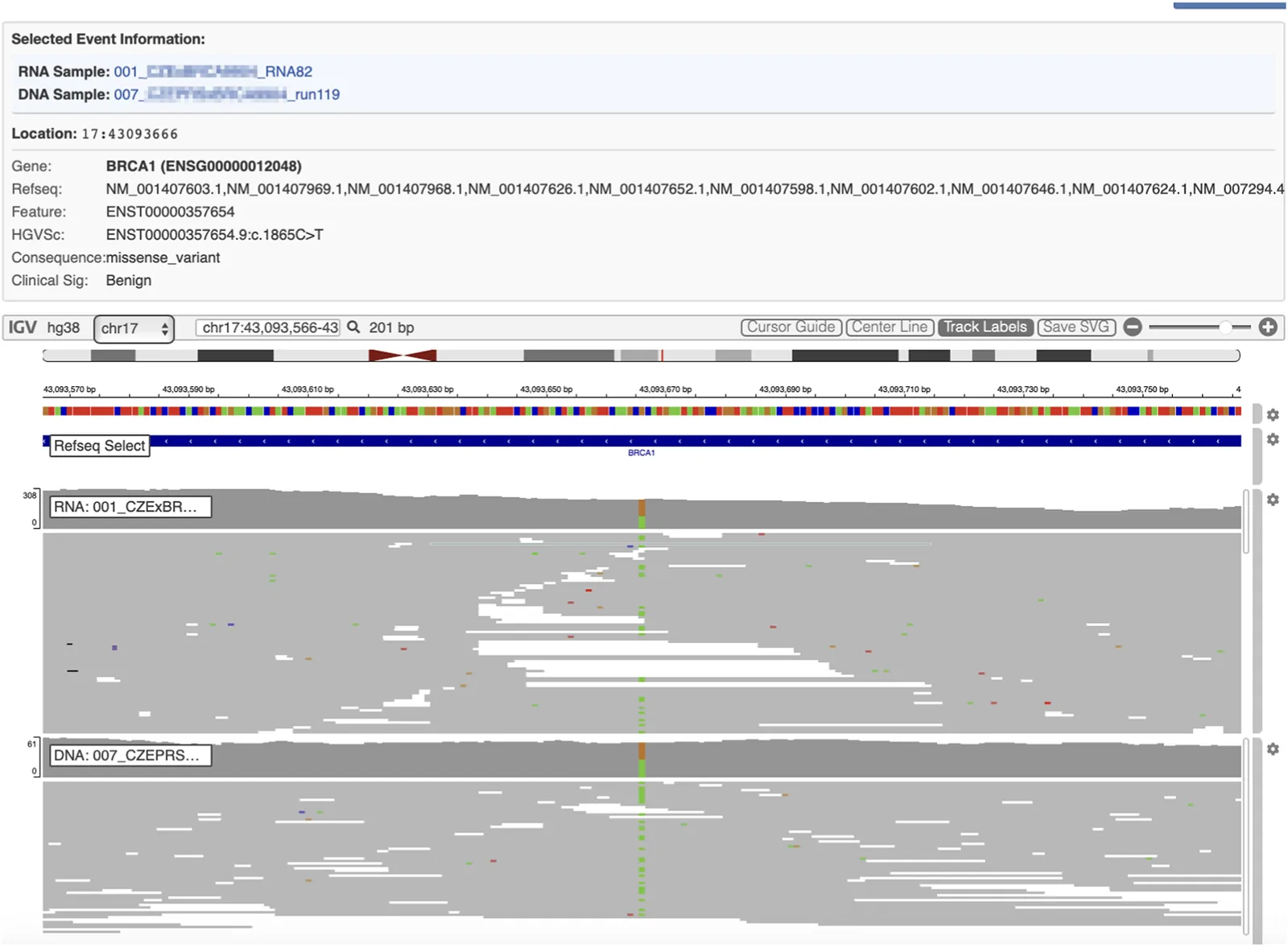
Parallel IGV visualization of a selected variant in Patient View. Upon selection of a variant, matched RNA (upper panel) and DNA (lower panel) BAM file windows are loaded from the NGS analyses (here illustrated for an individual harboring the *BRCA1* missense variant c.1865C>T).

This integrated view provides direct comparisons of critical quality parameters, including total depth (DP) for coverage assessment and genotype quality (GQ) for exclusion of low-quality calls. Allelic depth (AD) and variant allele frequency (VAF) enable the direct DNA-to-RNA VAF comparison, facilitating detection of allelic imbalances such as ASE and NMD.

The application embeds the Integrative Genomics Viewer (IGV) through igvShiny (Shannon et al., 2025) for visual validation of matched DNA and RNA BAM slices, with genomic coordinates set automatically based on the inspected variant locus.

#### 3.2.2 Functional data integration

The application dynamically integrates several distinct layers of functional evidence (Figure 2).

- called and thoroughly annotated DNA/RNA variants;
- allele-specific expression (ASE);
- read counts (gene expression) with GTEx reference data;
- expression outliers (computed via OUTRIDER); and
- aberrant splicing events (computed via FRASER) with corresponding Sashimi plots (available in Patient View only) of the affected sample and three randomly selected reference samples.

To aid variant interpretation, the platform embeds external APIs, including multi-tissue expression profiling from the GTEx Portal (V8) (GTEx Consortium, 2013).

#### 3.2.3 Bidirectional multiomics evidence joining

A major challenge in multiomics diagnostics is accurately linking DNA variants to RNA outliers. To resolve this, we implemented a bidirectionally reactive filtering engine. Selecting a DNA or RNA variant (Figure 2) instantly filters all RNA outlier tables to show only events mechanistically linked to that variant. Conversely, selecting an RNA outlier instantly queries the DNA variant pool for a potential causal variant. This filtering can be applied at the variant level or globally across all evidence layers, ensuring consistent results throughout.

For expression-based evidence (ASE, Read Counts, and OUTRIDER), data joining relies primarily on unique Ensembl Gene IDs (ENSG). Importantly, our platform is designed to handle incomplete variant annotations. If a variant caller fails to assign a gene ID to a regulatory variant, the platform employs a coordinate fallback strategy utilizing GenomicRanges, a Bioconductor package (Lawrence et al., 2013). It queries a reference transcriptome (GTF) to establish the true physical boundaries of the expression outlier’s gene. Any unannotated variant falling within this spatial window for the affected individual is successfully rescued and linked to the expression outlier.

#### 3.2.4 Biologically bounded integration of splicing outliers

Splicing aberrations present a unique integration challenge, as a causal spliceogenic variant may reside at canonical splice sites (±1, 2), within an adjacent exon, or deep intronic sequences.

When a FRASER splicing outlier is selected, the application queries the reference GTF to identify the exact physical coordinates of the nearest upstream (donor) and downstream (acceptor) exons adjacent to the aberrant junction. The platform dynamically constructs a custom, continuous genomic window that precisely spans from the start of the upstream exon to the end of the downstream exon.

By enforcing spatial intersections against this biologically expanded window, the application reliably captures causative splice-site variants, deep intronic cryptic variants, and exonic regulatory disruptions, without relying on rigid distance buffers that risk capturing adjacent and irrelevant gene variants or missing some of the causative ones.

In Patient View, clicking on a splice junction of interest triggers a Sashimi plot alongside an IGV window displaying the corresponding genomic range.

### 3.3 The *paRDal* framework in real-life examples

Below, we present several cases illustrating the utility of the *paRDal* framework across specific functionalities, selected from analyses of individuals undergoing genetic testing for hereditary cancer predisposition.

#### 3.3.1 Splicing landscape in a set of 204 individuals tested for hereditary cancer predisposition

We have analyzed the splicing landscape in 204 individuals who underwent germline genetic testing for hereditary cancer predisposition. Extraction of SNPs from the original dataset (excluding indels and frameshift variants) yielded 18,804 variants; regulatory variants were also excluded. We then applied the same quality and frequency thresholds (minimum depth >10, maximum possible genotype quality GQ = 99 in either DNA or RNA, and population frequency <0.01 or NA) but explicitly omitted the ClinVar significance filter. This parallel filtering strategy limited the dataset to 5,034 variants, which serves as the basis for the subsequent splicing analysis (Figure 4). Of these, 292 (5.8%) (Figure 4A) mapped to splice regions (in the vicinity of the splice site or in the polypyrimidine tract); however, most were absent from ClinVar. The largest proportion of variants with high SpliceAI scores and positive FRASER hits were classified as pathogenic (Figures 4A, 5), with the majority residing at donor or acceptor sites. In contrast, variants classified as likely benign or VUS exhibited moderate to high SpliceAI with a positive FRASER hit predominantly located in the polypyrimidine tract. As shown in Figure 4B, many FRASER hits lacked an associated causal variant, suggesting causative variants may reside in deep intronic sequences or regulatory exonic elements. This explanation is plausible, since this analysis only featured variants in the splice region; however, Supplementary Table S4 indicates that a substantial portion of these unassociated hits appear to be mapping artifacts.

**FIGURE 4:**
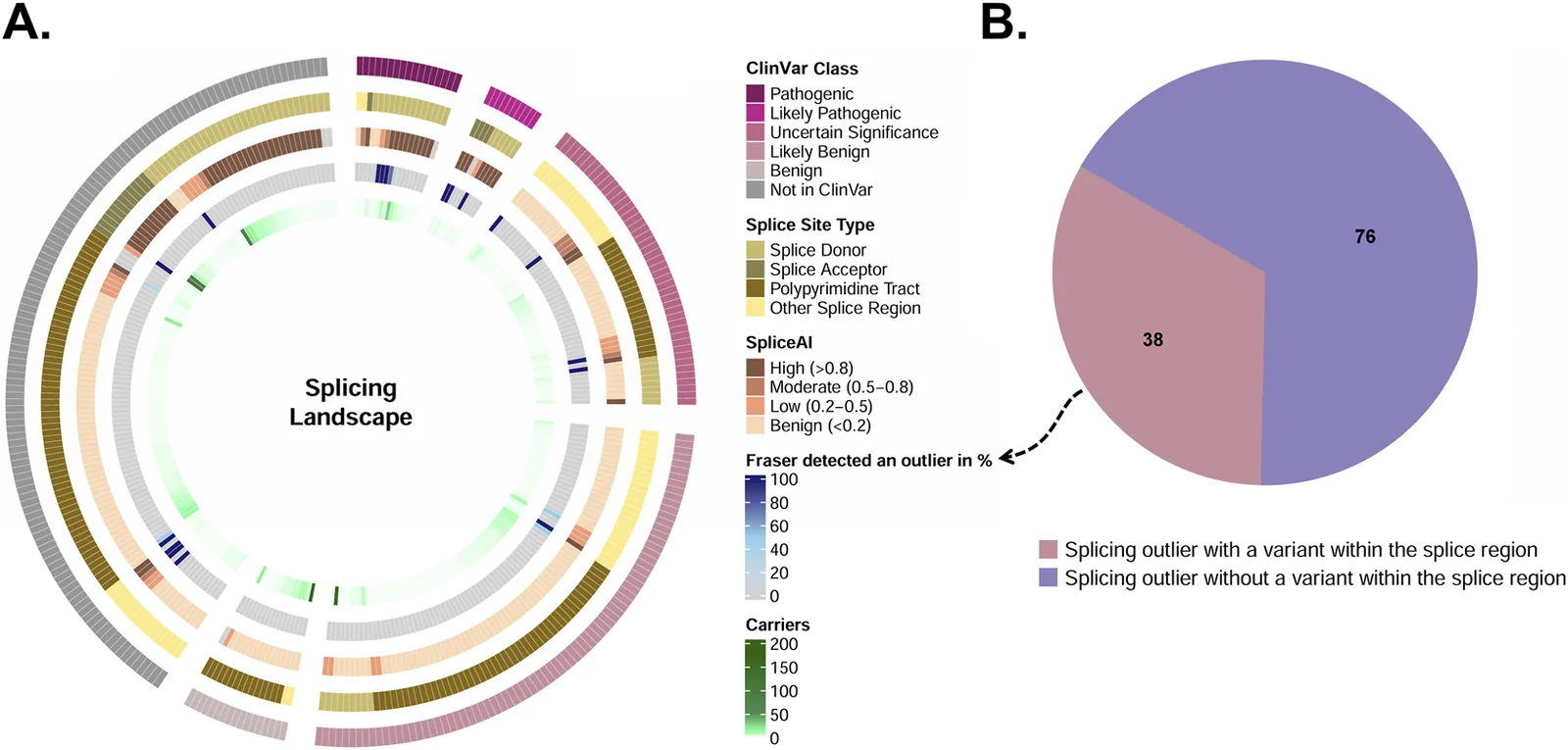
Splicing landscape of 292 unique variants mapping to splice regions **(A)**, derived from a quality-filtered set of 5,034 variants in 204 individuals tested for hereditary cancer predisposition. FRASER analysis **(B)** of the same sample set revealed 114 positive hits (splicing outliers), of which 38 (33%) were linked to a causal variant located within the splice region. Splicing outliers with high SpliceAI scores were enriched among pathogenic/likely pathogenic ClinVar variants; however, they were also identified among VUS and few likely benign variants. Eight (7%) FRASER-positive hits in 13 individuals scored to variants not described in ClinVar.

**FIGURE 5:**
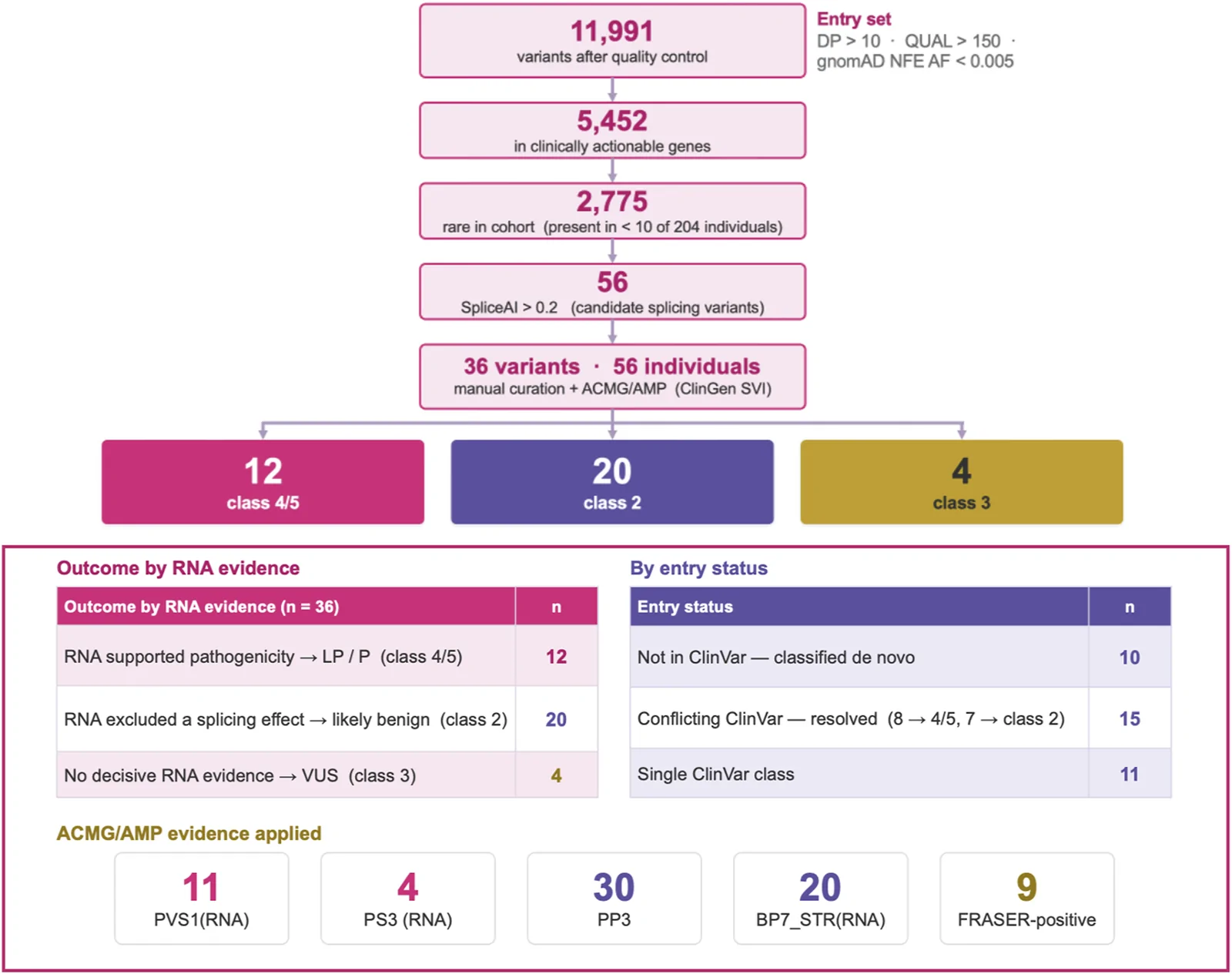
Cohort filtering funnel and RNA-informed ACMG/AMP classification of candidate splicing variants. Variants were filtered from the full dataset (11,991 quality-controlled variants; DP > 10, QUAL >150, gnomAD non-Finnish European allele frequency <0.005) through clinically actionable genes (5,452), cohort frequency (2,775; present in fewer than 10 of 204 individuals) and SpliceAI > 0.2 (56), yielding 36 candidate splicing variants across 56 carriers, which were evaluated using *paRDal* RNA evidence under the ClinGen Sequence Variant Interpretation (SVI) splicing recommendations. RNA evidence supported pathogenicity in 12 variants [PVS1(RNA) and/or PS3; class 4/5] and excluded a predicted splicing effect in 20 [BP7_STR(RNA); class 2], while four were classified as VUS. Lower panels summarize outcomes by RNA evidence and by prior ClinVar entry status (novel, conflicting, or single class), and the ACMG/AMP evidence codes applied across the cohort. Class: 1, benign; 2, likely benign; 3, VUS; 4, likely pathogenic; 5, pathogenic.

Of 114 splicing outliers detected across the cohort, 94 fell in established, non-overlapping genes. These collapsed to 67 distinct events, of which 28 were confirmed as genuine aberrant splicing on manual review. The biologically bounded window retrieved a candidate DNA variant for 23 events (82%), with a maximum of two accompanying non-causal variants per retrieval and a median of 0. Of the 32 distinct events with no candidate variant, only five were genuine aberrant splicing, indicating that most unexplained outliers are technical rather than biological in origin (Figure 6; Supplementary Table S4).

**FIGURE 6:**
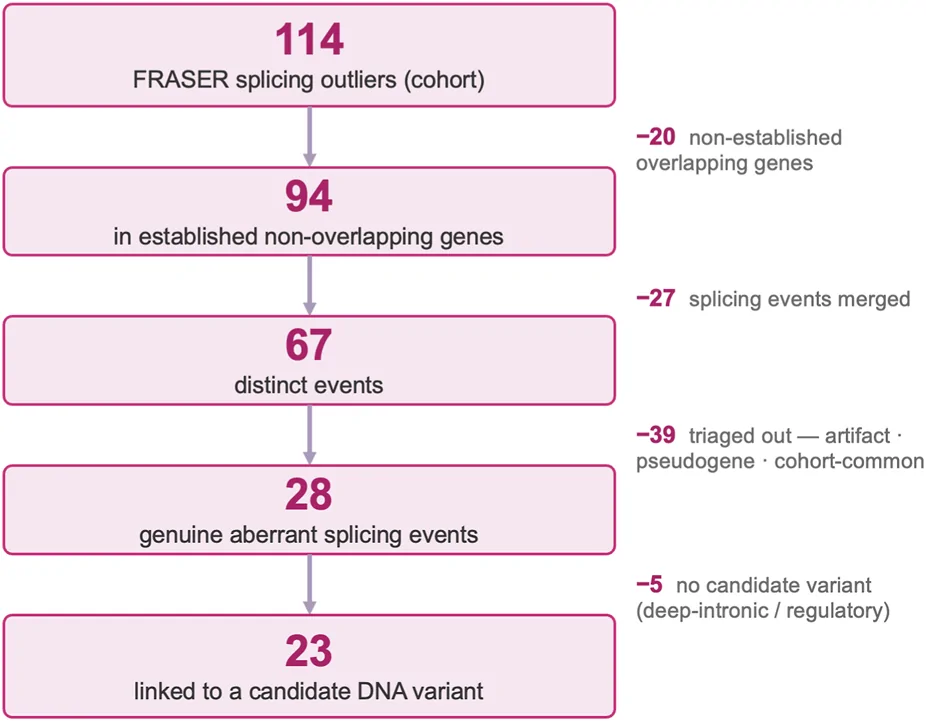
Curation burden and RNA-to-DNA linkage for FRASER splicing outliers. Of 114 FRASER splicing outliers detected across the cohort, 94 fell within established, non-overlapping genes. These collapsed to 67 distinct splicing events after merging exon-skipping and intron-retention junctions belonging to the same event; manual review confirmed 28 as genuine aberrant splicing events, while 39 were resolved as mapping artifacts, pseudogene alignments, or cohort-common splicing. The biologically bounded genomic window (spanning the upstream to downstream exon) retrieved a candidate DNA variant for 23 of the 28 genuine events (82%); the remaining five had no candidate variant and are consistent with a deep-intronic or regulatory cause.

To quantify the workflow’s output across the cohort, we traced all variants from quality control to final interpretation (Figure 5; Supplementary Table S5). Of 11,991 variants that passed quality control and were present in less than 0.5% of the European non-Finnish population, 5,452 lay in clinically actionable genes, 2,775 were rare in the cohort (present in <10/204 individuals) and 56 had SpliceAI >0.2; after manual curation, 36 unique candidate splicing variants in 56 carriers were evaluated with *paRDal* RNA evidence under the ClinGen SVI framework (Walker et al., 2023). RNA evidence supported pathogenicity in 12 variants [PVS1(RNA) or PS3; class 4/5] and excluded a predicted splicing effect in 20 [BP7_STR(RNA); class 2], while four remained VUS. Ten variants were absent from ClinVar and classified *de novo*, and 15 resolved conflicting ClinVar entries, indicating that *paRDal* predominantly resolves uncertain or previously unclassified variants rather than confirming established calls. Per-variant results and criterion assignments are provided in Supplementary Table S5.

#### 3.3.2 Detection of complex splicing disruptions caused by variants with inconclusive evidence (*ATM*:c.6197A>G)

A carrier of a rare variant affecting the penultimate nucleotide in exon 42 (ENST00000675843:c.6197A>G; Figure 7A, lower panel) exhibited skipping of exon 42 (E42Δ) in *ATM*. While predicted to cause a missense amino acid change (p.Gln2066Arg), this extremely rare variant (gnomAD NFE MAF = 0.0000009) is currently classified as a VUS in ClinVar. Integrated analysis using *paRDal* revealed a severe failure of the splicing machinery that *in silico* models failed to predict. The variant weakened the donor splice site, leading to the use of an upstream splice donor with *ΔΨ* = 0.11. Reference samples showed only a negligible amount of exon skipping (Figure 7B).

**FIGURE 7:**
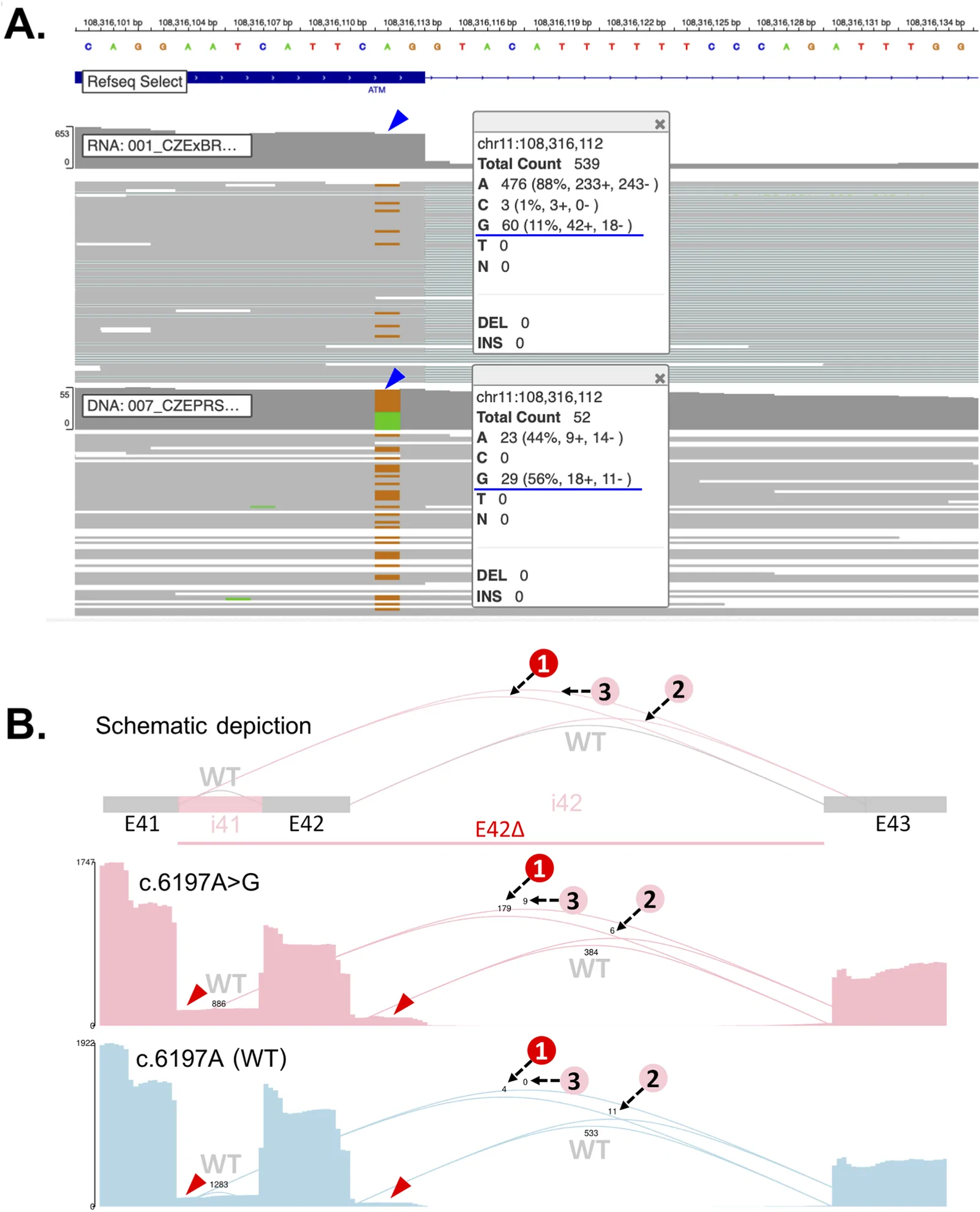
Visualization of the *ATM* c.6197A>G variant (**(A)**; blue arrowhead) in IGV showing parallel RNA (upper panel) and DNA (lower panel) NGS data from a carrier in *paRDal*. RNA VAF is substantially reduced relative to DNA (VAF% underlined in blue). Sashimi plots **(B)** for the variant carrier and a wild-type (WT) reference sample revealed skipping of E42 (①1; 179 vs. 4 events in the carrier and WT sample, respectively) accompanied by enhanced retention of flanking introns (red arrowheads). Minor alternative splicing events (②; ③) did not differ significantly between carrier and WT sample (6 vs. 11 and 9 vs. 0 events, respectively).

This was accompanied by the retention of both flanking introns (i41 and i42; Figure 7B), present in substantially more reads (*ΔΨ* = −0.2 for i41 and *ΔΨ* = −0.27 for i42).

The variant allele was significantly underrepresented in RNA (VAF 0.11) compared to DNA (VAF 0.56). While ASE analysis lacked statistical power due to limited heterozygous SNPs, the allelic imbalance is clear. The variant is predominantly found in unprocessed pre-mRNA, contributing to only 4% of reads with wild-type junctions and less than 10% to the overall wild-type mRNA pool.

Although the relatively low ΔΨ (0.11) might initially suggest minimal exon skipping, this metric is confounded by nonsense-mediated decay (NMD). The strong allelic imbalance and the absence of the variant allele in the isoforms carrying the wild-type junctions mean that the exon-skipped isoforms are rapidly degraded by NMD, masking the true severity of the splicing defect and effectively depleting the aberrant transcripts.

#### 3.3.3 Elucidation of the impact of deep intronic variants (*ATM*:c.1899-123A>G)

A rare deep intronic variant in *ATM* (ENST00000675843:c.1899-123A>G), located 123 bp upstream of exon 13, was prioritized due to a high SpliceAI score (>0.5), suggesting cryptic splice site activation. FRASER detected a mixture of pseudo-exonizations (one dominant with *ΔΨ* = −0.28) with three alternative acceptors, and varying degrees of exon elongation (one dominant *ΔΨ* = 0.17), confirmed by IGV visual inspection and Sashimi plots (Figure 8).

**FIGURE 8:**
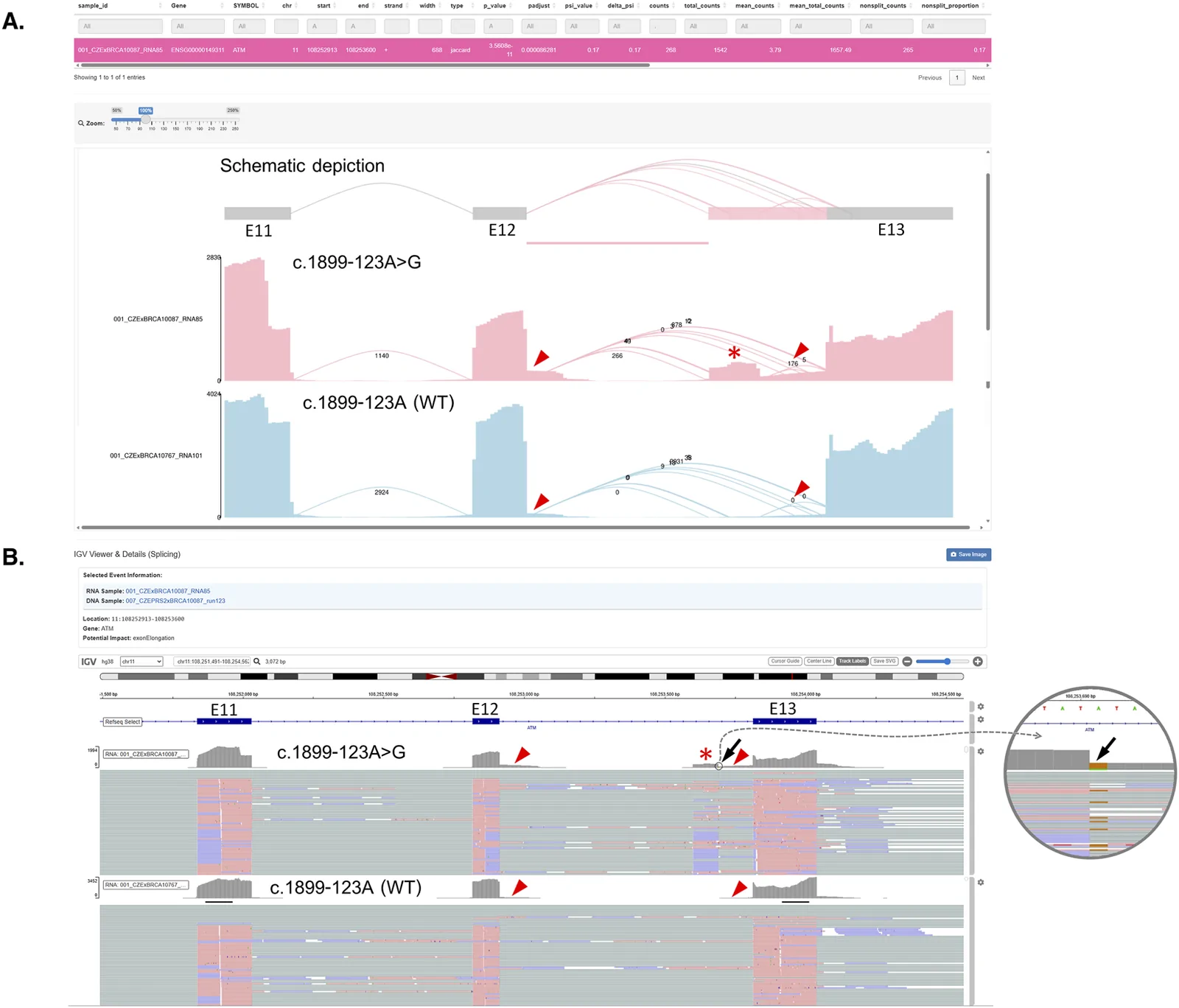
Sashimi plots **(A)** and IGV view **(B)** for an aberrant splicing event caused by *ATM*:c.1899-123A>G in *paRDal*, shown alongside a wild-type reference. Both panels show pseudo-exonization (*) of in an intrinsic segment of the intron 12 and intron 12 retention (red arrowheads). The c.1899-123A>G variant (**(B)**; black arrow) is shown at higher magnification (right inset) to illustrate increased proportion of the variant allele within the retained intron sequence.

Exon-restricted ASE showed no significant allelic imbalance in the mature transcript pool, providing no direct evidence of transcript degradation. At the deep-intronic variant position, covered only by intron-retaining reads, the variant allele predominated (0.86 vs. 0.4 VAF in DNA), indicating allele-specific intron retention.

#### 3.3.4 Two-pass STAR+WASP mapping for rescue of novel splice-junctions (*APC*:c.1627-8A>G)

FRASER detected a complex splicing pattern, described as a (partial) intron retention with *ΔΨ* = −0.26 of intron 13 in *APC*, involving two competing alternative acceptor sites. One site, a 7-bp exon elongation supported by only five reads, was linked to a variant in the polypyrimidine tract, while a seemingly distinct, better-supported non-canonical junction (46 reads) was detected 12 bp downstream (Figure 9a), adjacent to a common synonymous polymorphism (ENST00000257430:c.1635G>A, MAX_AF = 0.84).

**FIGURE 9:**
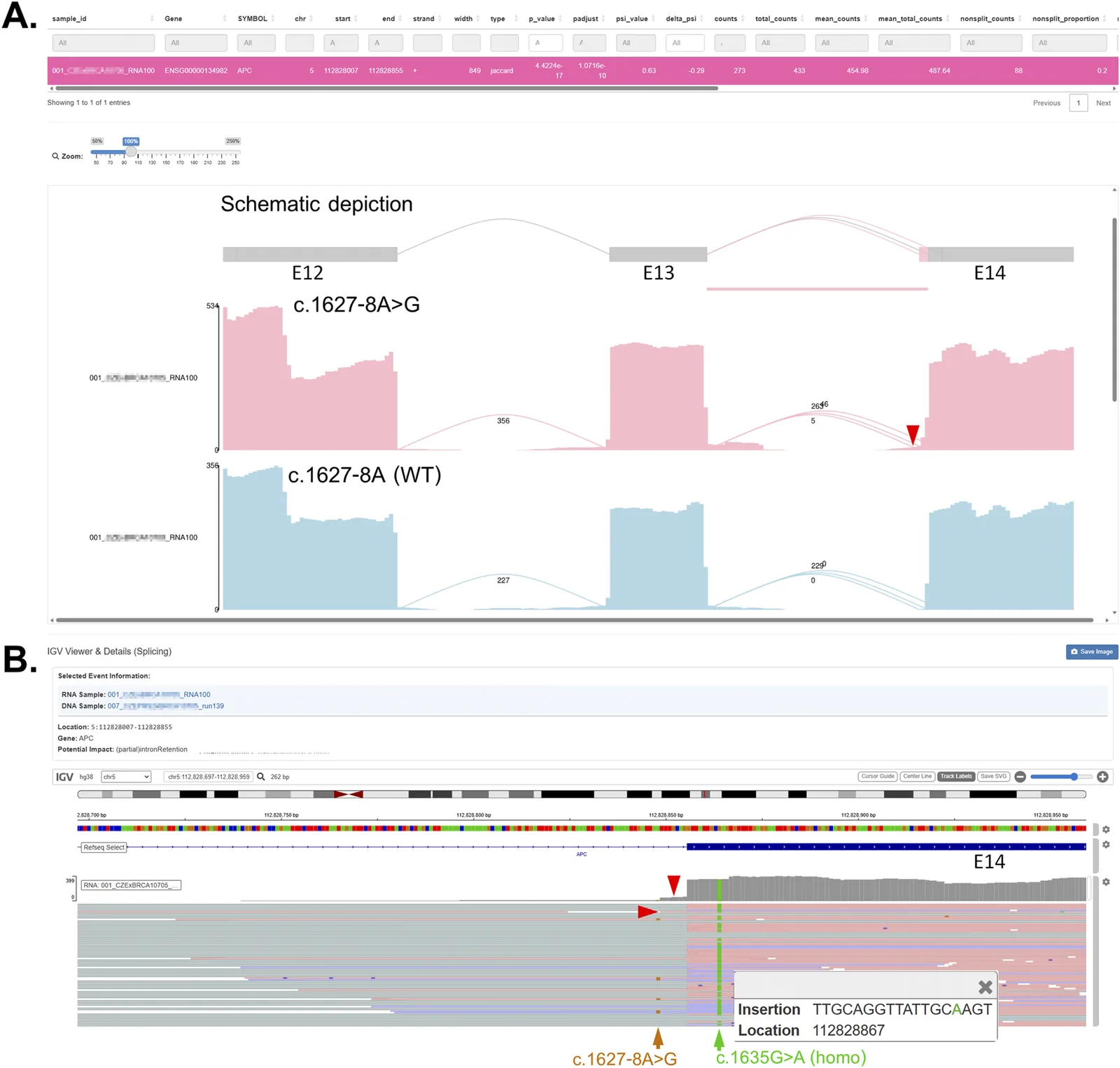
Sashimi plots **(A)** for *APC*:c.1627-8A>G. The carrier RNA sample depicts two novel aberrant acceptor sites absent from the wild-type (WT) reference, producing partial (7 bp) exon elongation (red arrowhead). The IGV plot **(B)** of the carrier RNA sample shows a small number of NGS reads spanning the c.1627-8A>G variant in unprocessed RNA, common homozygous polymorphism c.1635G>A, and, three bases downstream, an erroneously mapped 18-nucleotide insertion representing the retained acceptor site sequence of intron 13 (correctly mapped only in the read highlighted by red horizontal arrowhead).

Despite a SpliceAI score of 0.99 for the polypyrimidine tract variant, visual inspection identified the 46-read junction as an alignment artifact; the c.1635G>A mismatch introduced a reference bias in which the associated mismatch penalty exceeded aligner tolerance during first-pass STAR mapping, causing these reads to be flagged as an 18-bp insertion (Figure 9b). Recognizing that these 46 reads and the original 5 junction reads represented the same biological event, we unified the signal into a single 7-bp exon elongation with 51 total supporting reads–a ten-fold improvement achieved through two-pass STAR+WASP realignment. Although ASE was detected, the low SNP density (2 SNPs, heterogeneity = 1) renders these results unreliable. Regardless, the 7-bp elongation is predicted to introduce a PTC and trigger NMD, supporting the application of PVS1(RNA) (or PP3) criterion.

To further evaluate the impact of two-pass alignment on splice junction detection sensitivity, we analyzed uniquely mapped read coverage across four clinically actionable genes (*APC*, *ATM*, *BRCA1*, and *MSH3*) in a single run of 204 individuals (Figure 10). On average, the two-pass strategy rescued spanning reads for 23% of splice junctions missed during the initial pass, while only 1.7% of reads were lost (in fact, they were not depleted but transitioned from uniquely mapping reads to multi-mappers). To further evaluate the utility of two-pass alignment, we performed a precision analysis on the rescued reads (Figure 11). This demonstrated the efficacy of two-pass mapping for novel junction discovery, yielding 488,711 unannotated junctions. The vast majority of these novel junctions feature the canonical GT-AG intron motif and are highly recurrent across multiple individuals.

**FIGURE 10:**
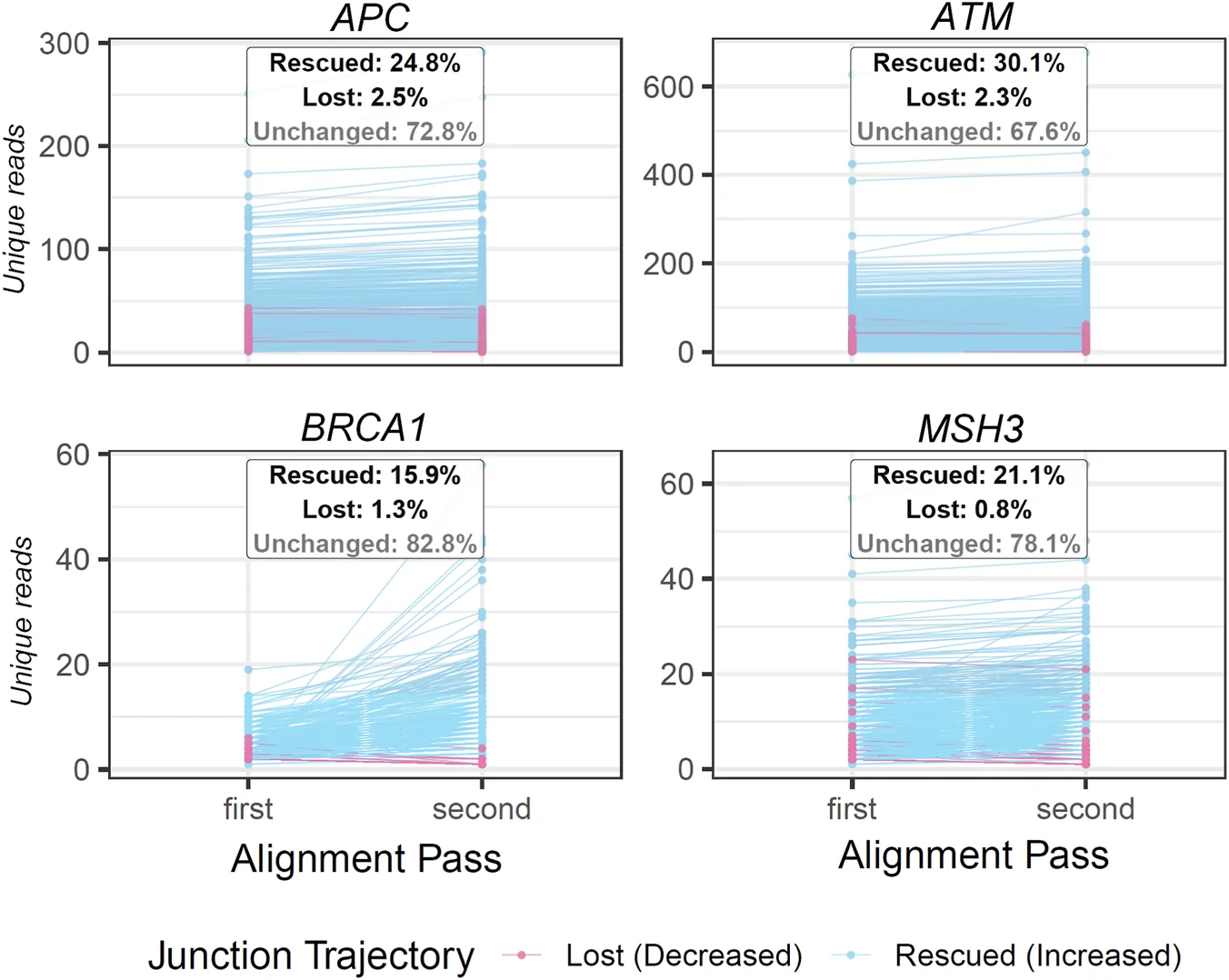
Rescue of novel splice junctions by two-pass STAR alignment across four clinically actionable multi-exon genes. The graphs show the number of novel splice junctions after the first (left) and second (right) STAR pass across 204 samples. Junctions newly detected in the second pass and unchanged junctions are not depicted.

**FIGURE 11:**
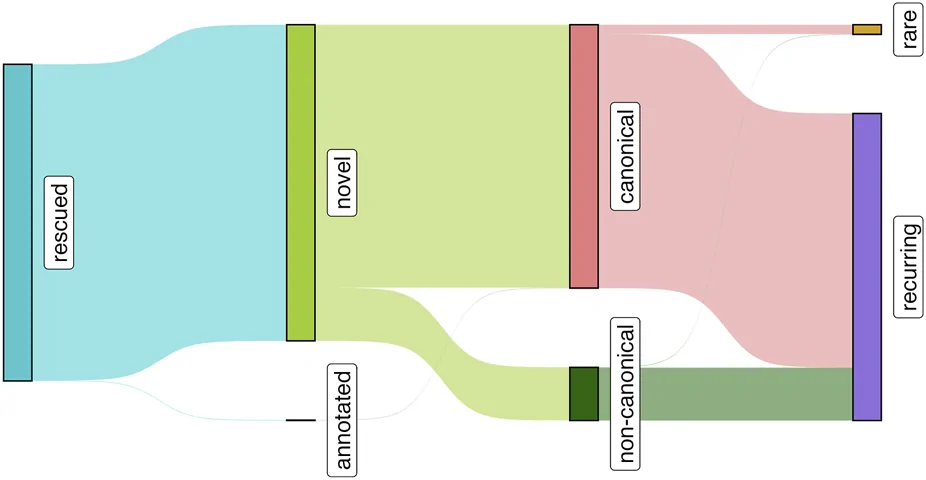
Precision analysis of the two-pass rescue. The plot illustrates 489,444 unique rescued reads (detected during the second pass) across 204 samples. Only 733 are annotated in ENSEMBL, while 488,711 are novel junctions, highlighting the efficacy of STAR two-pass mapping for novel junction discovery. Among the novel junctions, 406,414 feature the canonical GT-AG intron motif, and 82,297 exhibit non-canonical motifs. The majority of all detected junctions (392,755) are recurrent, appearing in more than two individuals.

#### 3.3.5 A cautionary tale: identification and curation of an *APC* promoter variant in siblings with GAPPS syndrome

In two siblings, both diagnosed with gastric adenocarcinoma and proximal polyposis of the stomach (GAPPS), *paRDal* identified a regulatory variant (ENSR5_6H8TT4, g.112707527T>C, c.-191T>C) within the *APC* promoter region. Although isolated in a non-coding regulatory element lacking standard gene-level annotation, the framework’s integrated table-joining strategy successfully linked these variants to *APC*, demonstrating *paRDal’*s ability to capture the impact of regulatory variants.

After restricting ASE to exonic positions, *APC* showed no significant allelic imbalance. Manual review of the parallel DNA/RNA table, per-patient metrics, and matched BAMs in IGV confirmed too few informative sites for a reliable estimate. No expression outlier was detected.

Despite the established pathogenicity of *APC* promoter variants in GAPPS syndrome (Foretová et al., 2019), the lack of informative heterozygous sites precluded detection of a biologically meaningful ASE. This case highlights the importance of the *paRDal* interactive interface, which enables clinicians to recognize the limitations of automated outputs and conduct the manual investigation necessary to confirm a variant’s functional impact.

## 4 Discussion

*paRDal* is an integrated clinical bioinformatics framework that operationalizes the transition between computational predictions (ACMG/AMP PP3 criterion) and functional clinical evidence (PVS1(RNA)). Demonstrated on an internal clinical cohort of 204 individuals and public reference datasets (Supplementary Table S5; Figure 5), *paRDal* unifies DNA/RNA variant calling and annotation with advanced transcriptomic analyses, including allele-aware two-pass alignment, expression outlier detection, aberrant splicing identification, and allele-specific expression, facilitating the application of ACMG/AMP criteria and accelerating VUS resolution in routine germline diagnostics.

Despite the potential of RNA NGS to resolve VUS (Horton et al., 2024; Rhine et al., 2018; Li et al., 2016; Buzby et al., 2017; Evans et al., 2018; Perrin-Vidoz et al., 2002; Brnich et al., 2019), its clinical adoption remains hampered by a lack of standardized bioinformatics workflows (Supplementary Figure S1, displays feature matrix, does not represent head-to-head evaluation). Existing frameworks suffer from fragmentation or narrow analytical focus, delegating raw RNA NGS preprocessing to the user and thereby compromising technical reproducibility and potentially diagnostic yield. Furthermore, established tools (Yépez et al., 2021; Maassen et al., 2025) typically rely on a unidirectional trajectory from outliers to the potentially causal variants. To address these systemic bottlenecks, *paRDal* offers optimized raw data pre-processing and employs a bidirectionally reactive engine, allowing geneticists to initiate queries from a candidate DNA/RNA VUS or, conversely, to anchor analysis on any observed transcriptomic signal and trace it back to a candidate variant.

While frameworks like DROP and the framework of Maassen et al. (2026) provide robust downstream analysis, they either delegate the critical pre-processing of raw RNA NGS FASTQs to the user or do not optimize those steps for downstream analysis. *nf-core* frameworks (Ewels et al., 2020) address only isolated analytical components, requiring manual synthesis of outputs. *paRDal* standardizes the entire trajectory from raw FASTQ processing to the final interactive application, implementing two critical mapping advancements: two-pass STAR alignment and WASP reference-allele bias correction, high-fidelity steps that, while individually addressed by specific modules (e.g., *nf-core* rnavar), are routinely omitted from standard pipelines (Figure 9).

In contrast to most available RNA NGS frameworks, which either do not integrate DNA variants or use them solely as a basis for ASE analysis, *paRDal* employs DNA/RNA variant calling primarily for variant exploration while also leveraging VCFs for sample authentication to exclude sample swaps.

While diagnostic prioritization increasingly relies on splicing, expression, and ASE as primary drivers, outlier-detection tools FRASER and OUTRIDER frequently fail to resolve VUS in isolation (Yépez et al., 2022; Jaramillo Oquendo et al., 2024; De Cock et al., 2025; Maassen et al., 2026). Statistical significance alone often remains inconclusive even for known splice-altering variants; the diagnostic utility of expression outliers is primarily limited to structural variants, and ASE rarely serves as a standalone diagnostic driver. Our findings demonstrate that successful variant classification requires interpreting outlier detection and ASE alongside variant quality metrics, *in silico* predictions, and visualization, rather than treating them as independent metrics. Manual curation remains essential when a variant is suspected to alter the transcriptomic profile (De Cock et al., 2025) (e.g., a splice-site variant should be evaluated in IGV, even in the absence of significant FRASER hits). *paRDal* specifically enables both DNA-centric (from a variant to a functional effect) and RNA-centric (from an observed effect to a causal variant) analytical perspectives. By consolidating conflicting scores, annotations, visual evidence, and per-sample metrics into a single interface, *paRDal* enables the time-efficient application of the PVS1(RNA) ACMG/AMP criterion, eliminating the need for manual cross-referencing and multi-step BAM inspection.

*paRDal* outputs feed the ACMG/AMP workflow at the point where prediction is the weakest: an observed transcript consequence is a direct measurement of what the *in silico* tools underlying PP3 can only predict. They serve as evidence for classification, not as the classification itself. Splicing outlier metrics such as ΔΨ describe deviation in the usage of individual junctions across the whole transcript pool; a single aberration is typically distributed across several such events and is not equivalent to the fraction of aberrant transcripts. Interpretation, therefore, requires the combined evidence: splicing, ASE, expression, and gene-specific transcript architecture, and the assignment of PVS1(RNA) strength under the ClinGen SVI framework (Walker et al., 2023) remains the responsibility of the interpreting laboratory.

*paRDal* is subject to several biological and computational limitations. The workflow currently applies predefined parameters and thresholds optimized specifically for Mendelian diagnostics, which may require adjustment for broader research contexts. Computationally, the autoencoders used for the outlier detection perform optimally with a minimum cohort size of 60 samples (Brechtmann et al., 2018), a requirement that can be mitigated by utilizing tissue-matched public datasets as a background reference. Biologically, diagnostic utility is limited by tissue-specific expression; although integration of GTEx data helps identify signals absent from the examined tissue. Highly efficient NMD may deplete aberrant isoforms, leaving some aberrant splicing events undetected. Future work will focus on integrating Pindel (Ye et al., 2009) and a dedicated CNV analysis module to address current limitations in large indel detection and provide a truly comprehensive multi-omics diagnostic solution.

## Data Availability

The datasets presented in this article are not publicly available. Paired germline DNA and RNA data are inherently identifiable, and the informed consent obtained from participants does not permit public deposition; open sharing would breach Regulation (EU) 2016/679 (GDPR) and Czech Act No. 110/2019 Coll. De-identified individual-level data are available to qualified researchers within the secure research environment of Charles University upon reasonable request, which should be directed to ZK (zdekleje@lf1.cuni.cz), and are subject to approval by the Ethics Committee of the General University Hospital in Prague and a data access agreement. All other data, including synthetic genotype data, are provided as Supplementary Material or in the unit test suite. The data for the minimal framework can be downloaded from https://www.ebi.ac.uk/ena/data/view/PRJEB31736 and the corresponding RNA can be accessed at https://www.ebi.ac.uk/biostudies/arrayexpress/studies/E-GEUV-3#o3.

## Supplementary material

SUPPLEMENTARY FIGURE S1 Feature matrix of state-of-the-art tools for integrated DNA/RNA analysis. Matrix compares *paRDal*’s features with currently available tools; however, it does not represent a head-to-head evaluation.

SUPPLEMENTARY TABLE S1 List of VEP annotation fields and plugins used in the *paRDal* pipeline, with the corresponding flag/plugin, functional group, and access date.

SUPPLEMENTARY TABLE S2 Third-party tools used in the *paRDal* pipeline, listed by category with version numbers and references.

SUPPLEMENTARY TABLE S3 Description of the DNA/RNA variant-level sequencing metrics displayed in the parallel analysis module, including per-sample presence status, quality scores, allelic depths, and VAFs.

SUPPLEMENTARY TABLE S4 FRASER splicing-outlier triage and RNA→DNA linkage. Summary sheet shows the triage funnel from raw outliers to genuine aberrant splicing events linked to candidate DNA variants; the *Distinct events* and *Junctions* sheets provide the underlying per-event and per-junction data.

SUPPLEMENTARY TABLE S5 Cohort filtering funnel and RNA-informed variant outcomes across 204 individuals. Summary sheet shows the stepwise filtering funnel; the *Variants* sheet lists prioritized variants with SpliceAI scores, FRASER support, and ACMG/AMP reclassification.

## References

[B1] Agiannitopoulos K. Pepe G. Papadopoulou E. Tsaousis G. N. Kampouri S. Maravelaki S. (2021). Clinical utility of functional RNA analysis for the reclassification of splicing gene variants in hereditary cancer. CGP 18 (3), 285–294. 10.21873/cgp.20259 33893081 PMC8126331

[B2] Auton A. Brooks L. D. Durbin R. M. Garrison E. P. Kang H. M. Korbel J. O. (2015). A global reference for human genetic variation. Nature 526 (7571), 68–74. 10.1038/nature15393 26432245 PMC4750478

[B3] Brechtmann F. Mertes C. Matusevičiūtė A. Yépez V. A. Avsec Ž. Herzog M. (2018). OUTRIDER: a statistical method for detecting aberrantly expressed genes in RNA sequencing data. Am. J. Hum. Genet. 103 (6), 907–917. 10.1016/j.ajhg.2018.10.025 30503520 PMC6288422

[B4] Brnich S. E. Abou Tayoun A. N. Couch F. J. Cutting G. R. Greenblatt M. S. Heinen C. D. (2019). Recommendations for application of the functional evidence PS3/BS3 criterion using the ACMG/AMP sequence variant interpretation framework. Genome Med. 12 (1), 3. 10.1186/s13073-019-0690-2 31892348 PMC6938631

[B5] Broad Institute (2019). Picard Toolkit. Broad Institute. GitHub repository 2019.

[B6] Burke W. Parens E. Chung W. K. Berger S. M. Appelbaum P. S. (2022). The challenge of genetic variants of uncertain clinical significance: a narrative review. Ann. Intern Med. 175 (7), 994–1000. 10.7326/m21-4109 35436152 PMC10555957

[B7] Buzby J. S. Williams S. A. Schaffer L. Head S. R. Nugent D. J. (2017). Allele-specific wild-type TP53 expression in the unaffected carrier parent of children with Li-Fraumeni syndrome. Cancer Genet. 211, 9–17. 10.1016/j.cancergen.2017.01.001 28279309 PMC5347445

[B8] Černá M. Šťastná B. Pešek P. Ptáčková T. Janatová M. Soukupová J. (2025). Semi-automated RNA isolation from tempus blood RNA tubes using the magcore plus II instrument. Folia Biol. (Praha) 71 (2), 88–94. 10.14712/fb2025071020088 40627838

[B9] Černá M. Matějková K. Ptáčková T. Nehasil P. Mihalová R. Veselá K. (2026). Short-term lymphocyte culture improves the diagnostic yield of targeted RNA NGS in cancer predisposition testing. Folia Biol. (Praha) 72 (1), 16–26. 10.14712/fb2026.0001 41979997

[B10] Chen E. Facio F. M. Aradhya K. W. Rojahn S. Hatchell K. E. Aguilar S. (2023). Rates and classification of variants of uncertain significance in hereditary disease genetic testing. JAMA Netw. Open 6 (10), e2339571. 10.1001/jamanetworkopen.2023.39571 37878314 PMC10600581

[B11] Cheng J. Novati G. Pan J. Bycroft C. Žemgulytė A. Applebaum T. (2023). Accurate proteome-wide missense variant effect prediction with AlphaMissense. Science 381 (6664), eadg7492. 10.1126/science.adg7492 37733863

[B12] Danecek P. McCarthy S. A. (2017). BCFtools/csq: haplotype-aware variant consequences. Bioinformatics 33 (13), 2037–2039. 10.1093/bioinformatics/btx100 28205675 PMC5870570

[B13] Dawes R. Bournazos A. M. Bryen S. J. Bommireddipalli S. Marchant R. G. Joshi H. (2023). SpliceVault predicts the precise nature of variant-associated mis-splicing. Nat. Genet. 55 (2), 324–332. 10.1038/s41588-022-01293-8 36747048 PMC9925382

[B14] De Cock L. D'Haenens E. Vantomme L. Backers L. Beyens A. Claes K. B. (2025). Cracking rare disorders: a new minimally invasive RNA-seq protocol. NPJ Genom Med. 10 (1), 45. 10.1038/s41525-025-00502-7 40436861 PMC12120114

[B15] den Dunnen J. T. Dalgleish R. Maglott D. R. Hart R. K. Greenblatt M. S. McGowan-Jordan J. (2016). HGVS recommendations for the description of sequence variants: 2016 update. Hum. Mutat. 37 (6), 564–569. 10.1002/humu.22981 26931183

[B16] Dobin A. Davis C. A. Schlesinger F. Drenkow J. Zaleski C. Jha S. (2013). STAR: ultrafast universal RNA-seq aligner. Bioinformatics 29 (1), 15–21. 10.1093/bioinformatics/bts635 23104886 PMC3530905

[B17] Dragoš V. Strojnik K. Klančar G. Škerl P. Stegel V. Blatnik A. (2022). Identification of spliceogenic variants beyond canonical GT-AG splice sites in hereditary cancer genes. Int. J. Mol. Sci. 23 (13), 7446. 10.3390/ijms23137446 35806449 PMC9267136

[B18] Dyer S. C. Austine-Orimoloye O. Azov A. G. Barba M. Barnes I. Barrera-Enriquez V. P. (2025). Ensembl 2025. Nucleic Acids Res. 53 (D1), D948–D957. 10.1093/nar/gkae1071 39656687 PMC11701638

[B19] Esposito D. Weile J. Shendure J. Starita L. M. Papenfuss A. T. Roth F. P. (2019). MaveDB: an open-source platform to distribute and interpret data from multiplexed assays of variant effect. Genome Biol. 20 (1), 223. 10.1186/s13059-019-1845-6 31679514 PMC6827219

[B20] Evans D. G. R. van Veen E. M. Byers H. J. Wallace A. J. Ellingford J. M. Beaman G. (2018). A dominantly inherited 5' UTR variant causing methylation-associated silencing of BRCA1 as a cause of breast and ovarian cancer. Am. J. Hum. Genet. 103 (2), 213–220. 10.1016/j.ajhg.2018.07.002 30075112 PMC6080768

[B21] Ewans L. J. Minoche A. E. Schofield D. Shrestha R. Puttick C. Zhu Y. (2022). Whole exome and genome sequencing in mendelian disorders: a diagnostic and health economic analysis. Eur. J. Hum. Genet. 30 (10), 1121–1131. 10.1038/s41431-022-01162-2 35970915 PMC9553973

[B22] Ewels P. A. Peltzer A. Fillinger S. Patel H. Alneberg J. Wilm A. (2020). The nf-core framework for community-curated bioinformatics pipelines. Nat. Biotechnol. 38 (3), 276–278. 10.1038/s41587-020-0439-x 32055031

[B23] Federici G. Soddu S. (2020). Variants of uncertain significance in the era of high-throughput genome sequencing: a lesson from breast and ovary cancers. J. Exp. Clin. Cancer Res. 39 (1), 46. 10.1186/s13046-020-01554-6 32127026 PMC7055088

[B24] Feng B. J. (2017). PERCH: a unified framework for disease gene prioritization. Hum. Mutat. 38 (3), 243–251. 10.1002/humu.23158 27995669 PMC5299048

[B25] Foretová L. Navrátilová M. Svoboda M. Grell P. Nemec L. Sirotek L. (2019). GAPPS - gastric adenocarcinoma and proximal polyposis of the stomach syndrome in 8 families tested at Masaryk Memorial Cancer Institute - prevention and prophylactic gastrectomies. Klin. Onkol. 32 (Suppl. 2), 109–117. 10.14735/amko2019S109 31409086

[B26] Frésard L. Smail C. Ferraro N. M. Teran N. A. Li X. Smith K. S. (2019). Identification of rare-disease genes using blood transcriptome sequencing and large control cohorts. Nat. Med. 25 (6), 911–919. 10.1038/s41591-019-0457-8 31160820 PMC6634302

[B27] Google (2026). Gemini 3.1 Pro.

[B28] GTEx Consortium (2013). The genotype-tissue expression (GTEx) project. Nat. Genet. 45 (6), 580–585. 10.1038/ng.2653 23715323 PMC4010069

[B29] Ha H. I. Ryu J. S. Shim H. Kong S. Y. Lim M. C. (2020). Reclassification of BRCA1 and BRCA2 variants found in ovarian epithelial, fallopian tube, and primary peritoneal cancers. J. Gynecol. Oncol. 31 (6), e83. 10.3802/jgo.2020.31.e83 33078592 PMC7593220

[B30] Horton C. Hoang L. Zimmermann H. Young C. Grzybowski J. Durda K. (2024). Diagnostic outcomes of concurrent DNA and RNA sequencing in individuals undergoing hereditary cancer testing. JAMA Oncol. 10 (2), 212–219. 10.1001/jamaoncol.2023.5586 37924330 PMC10625669

[B31] Huang K. L. Mashl R. J. Wu Y. Ritter D. I. Wang J. Oh C. (2018). Pathogenic germline variants in 10,389 adult cancers. Cell 173 (2), 355–370.e14. 10.1016/j.cell.2018.03.039 29625052 PMC5949147

[B32] Ioannidis N. M. Rothstein J. H. Pejaver V. Middha S. McDonnell S. K. Baheti S. (2016). REVEL: an ensemble method for predicting the pathogenicity of rare missense variants. Am. J. Hum. Genet. 99 (4), 877–885. 10.1016/j.ajhg.2016.08.016 27666373 PMC5065685

[B33] Jaganathan K. Kyriazopoulou Panagiotopoulou S. McRae J. F. Darbandi S. F. Knowles D. Li Y. I. (2019). Predicting splicing from primary sequence with deep learning. Cell 176 (3), 535–548.e24. 10.1016/j.cell.2018.12.015 30661751

[B34] Jaramillo Oquendo C. Wai H. A. Rich W. I. Bunyan D. J. Thomas N. S. Hunt D. (2024). Identification of diagnostic candidates in Mendelian disorders using an RNA sequencing-centric approach. Genome Med. 16 (1), 110. 10.1186/s13073-024-01381-w 39252027 PMC11382415

[B35] Kamps-Hughes N. Carlton V. E. H. Fresard L. Osazuwa S. Starks E. Vincent J. J. (2023). A systematic method for detecting abnormal mRNA splicing and assessing its clinical impact in individuals undergoing genetic testing for hereditary cancer syndromes. J. Mol. Diagn 25 (3), 156–167. 10.1016/j.jmoldx.2022.12.002 36563937

[B36] Kircher M. Witten D. M. Jain P. O'Roak B. J. Cooper G. M. Shendure J. (2014). A general framework for estimating the relative pathogenicity of human genetic variants. Nat. Genet. 46 (3), 310–315. 10.1038/ng.2892 24487276 PMC3992975

[B37] Kleiblová P. Černá M. Zemánková P. Matějková K. Nehasil P. Hojný J. (2024). Parallel DNA/RNA NGS using an identical target enrichment panel in the analysis of hereditary cancer predisposition. Folia Biol. (Praha) 70 (1), 62–73. 10.14712/fb2024070010062 38830124

[B38] Kobayashi Y. Chen E. Facio F. M. Metz H. Poll S. R. Swartzlander D. (2024). Clinical variant reclassification in hereditary disease genetic testing. JAMA Netw. Open 7 (11), e2444526. 10.1001/jamanetworkopen.2024.44526 39504018 PMC11541632

[B39] Kuzbari Z. Bandlamudi C. Loveday C. Garrett A. Mehine M. George A. (2023). Germline-focused analysis of tumour-detected variants in 49,264 cancer patients: ESMO precision medicine working group recommendations. Ann. Oncol. 34 (3), 215–227. 10.1016/j.annonc.2022.12.003 36529447

[B40] Lappalainen T. Sammeth M. Friedländer M. R. t Hoen P. A. Monlong J. Rivas M. A. (2013). Transcriptome and genome sequencing uncovers functional variation in humans. Nature 501 (7468), 506–511. 10.1038/nature12531 24037378 PMC3918453

[B67] Lawrence M. Huber W. Pagès H. Aboyoun P. Carlson M. Gentleman R. (2013). Software for computing and annotating genomic ranges. PLoS Comput. Biol. 9 (8), e1003118. 10.1371/journal.pcbi.1003118 23950696 PMC3738458

[B41] Li J. Woods S. L. Healey S. Beesley J. Chen X. Lee J. S. (2016). Point mutations in exon 1B of APC reveal gastric adenocarcinoma and proximal polyposis of the stomach as a familial adenomatous polyposis variant. Am. J. Hum. Genet. 98 (5), 830–842. 10.1016/j.ajhg.2016.03.001 27087319 PMC4863475

[B42] Luo X. Xiao B. Liang L. Zhang K. Xu T. Liu H. (2025). Blood RNA-seq in rare disease diagnostics: a comparative study of cases with and without candidate variants. J. Transl. Med. 23 (1), 586. 10.1186/s12967-025-06609-w 40420094 PMC12105386

[B43] Maassen W. T. K. Johansson L. F. Charbon B. Hendriksen D. van den Hoek S. Slofstra M. K. (2025). MOLGENIS VIP: an end-to-end DNA variant interpretation pipeline for research and diagnostics configurable to support rapid implementation of new methods. NAR Genom. Bioinform. 7 (2), lqaf087. 10.1093/nargab/lqaf087 40585303 PMC12205968

[B44] Maassen W. T. K. Pape C. Urzua-Traslavina C. G. Niemeijer T. van Lieshout T. van der Molen M. (2026). Accelerating rare disease diagnostics by linking DNA and RNA through an explainable and interactive RNA-guided workflow. NAR Genom. Bioinform. 8 (1), lqag016. 10.1093/nargab/lqag016 41685349 PMC12891912

[B46] McLaren W. Gil L. Hunt S. E. Riat H. S. Ritchie G. R. Thormann A. (2016). The ensembl variant effect predictor. Genome Biol. 17 (1), 122. 10.1186/s13059-016-0974-4 27268795 PMC4893825

[B47] Mertes C. Scheller I. F. Yépez V. A. Çelik M. H. Liang Y. Kremer L. S. (2021). Detection of aberrant splicing events in RNA-seq data using FRASER. Nat. Commun. 12 (1), 529. 10.1038/s41467-020-20573-7 33483494 PMC7822922

[B48] Morales J. Pujar S. Loveland J. E. Astashyn A. Bennett R. Berry A. (2022). A joint NCBI and EMBL-EBI transcript set for clinical genomics and research. Nature 604 (7905), 310–315. 10.1038/s41586-022-04558-8 35388217 PMC9007741

[B49] Ng P. C. Henikoff S. (2001). Predicting deleterious amino acid substitutions. Genome Res. 11 (5), 863–874. 10.1101/gr.176601 11337480 PMC311071

[B50] Perrin-Vidoz L. Sinilnikova O. M. Stoppa-Lyonnet D. Lenoir G. M. Mazoyer S. (2002). The nonsense-mediated mRNA decay pathway triggers degradation of most BRCA1 mRNAs bearing premature termination codons. Hum. Mol. Genet. 11 (23), 2805–2814. 10.1093/hmg/11.23.2805 12393792

[B51] Rhine C. L. Cygan K. J. Soemedi R. Maguire S. Murray M. F. Monaghan S. F. (2018). Hereditary cancer genes are highly susceptible to splicing mutations. PLoS Genet. 14 (3), e1007231. 10.1371/journal.pgen.1007231 29505604 PMC5854443

[B52] Sha Y. Ortiz J. B. Bristow S. L. Loranger K. Meng L. Zhao X. (2025). Refining the interpretation of variants of uncertain significance in hereditary cancer screening through integrated RNA sequencing. Genet. Med. Open 3, 101914. 10.1016/j.gimo.2024.101914 39902189 PMC11788803

[B53] Shannon P. Gladki A. Scigocka K. (2025). igvShiny: a wrapper of integrative genomics viewer (IGV - an interactive tool for visualization and exploration integrated genomic data). Version 1.6.0.

[B54] Smedley D. Smith K. R. Martin A. Thomas E. A. McDonagh E. M. Cipriani V. (2021). 100,000 genomes pilot on rare-disease diagnosis in health care - preliminary report. N. Engl. J. Med. 385 (20), 1868–1880. 10.1056/NEJMoa2035790 34758253 PMC7613219

[B55] Soukupova J. Zemankova P. Lhotova K. Janatova M. Borecka M. Stolarova L. (2018). Validation of CZECANCA (CZEch CAncer paNel for Clinical Application) for targeted NGS-based analysis of hereditary cancer syndromes. PLoS One 13 (4), e0195761. 10.1371/journal.pone.0195761 29649263 PMC5896995

[B56] Tan A. Abecasis G. R. Kang H. M. (2015). Unified representation of genetic variants. Bioinformatics 31 (13), 2202–2204. 10.1093/bioinformatics/btv112 25701572 PMC4481842

[B57] Truty R. Ouyang K. Rojahn S. Garcia S. Colavin A. Hamlington B. (2021). Spectrum of splicing variants in disease genes and the ability of RNA analysis to reduce uncertainty in clinical interpretation. Am. J. Hum. Gen. 108 (4), 696–708. 10.1016/j.ajhg.2021.03.006 33743207 PMC8059334

[B58] van de Geijn B. McVicker G. Gilad Y. Pritchard J. K. (2015). WASP: allele-specific software for robust molecular quantitative trait locus discovery. Nat. Methods 12 (11), 1061–1063. 10.1038/nmeth.3582 26366987 PMC4626402

[B59] Van der Auwera G. A. Carneiro M. O. Hartl C. Poplin R. Del Angel G. Levy-Moonshine A. (2013). From FastQ data to high confidence variant calls: the genome analysis toolkit best practices pipeline. Curr. Protoc. Bioinforma. 43 (1110), 11.10.1–11.10.33. 10.1002/0471250953.bi1110s43 25431634 PMC4243306

[B60] Vasimuddin M. Misra S. Li H. Aluru S. (2019). Efficient architecture-aware acceleration of BWA-MEM for multicore systems. 2019 IEEE International Parallel and Distributed Processing Symposium (IPDPS), 314–324. 10.1109/IPDPS.2019.00041

[B61] Walker L. C. Hoya M. Wiggins G. A. R. Lindy A. Vincent L. M. Parsons M. T. (2023). Using the ACMG/AMP framework to capture evidence related to predicted and observed impact on splicing: recommendations from the ClinGen SVI splicing subgroup. Am. J. Hum. Genet. 110 (7), 1046–1067. 10.1016/j.ajhg.2023.06.002 37352859 PMC10357475

[B62] Wojcik M. H. Lemire G. Berger E. Zaki M. S. Wissmann M. Win W. (2024). Genome sequencing for diagnosing rare diseases. N. Engl. J. Med. 390 (21), 1985–1997. 10.1056/nejmoa2314761 38838312 PMC11350637

[B63] Ye K. Schulz M. H. Long Q. Apweiler R. Ning Z. (2009). Pindel: a pattern growth approach to detect break points of large deletions and medium sized insertions from paired-end short reads. Bioinformatics 25 (21), 2865–2871. 10.1093/bioinformatics/btp394 19561018 PMC2781750

[B64] Yépez V. A. Mertes C. Müller M. F. Klaproth-Andrade D. Wachutka L. Frésard L. (2021). Detection of aberrant gene expression events in RNA sequencing data. Nat. Protoc. 16 (2), 1276–1296. 10.1038/s41596-020-00462-5 33462443

[B65] Yépez V. A. Gusic M. Kopajtich R. Mertes C. Smith N. H. Alston C. L. (2022). Clinical implementation of RNA sequencing for Mendelian disease diagnostics. Genome Med. 14 (1), 38. 10.1186/s13073-022-01019-9 35379322 PMC8981716

[B66] Zheng X. Levine D. Shen J. Gogarten S. M. Laurie C. Weir B. S. (2012). A high-performance computing toolset for relatedness and principal component analysis of SNP data. Bioinformatics 28 (24), 3326–3328. 10.1093/bioinformatics/bts606 23060615 PMC3519454

