# Supplementary material for: paRDal: bioinformatics framework for a parallel RNA and DNA NGS analysis in hereditary cancer diagnostics
Source: Front Bioinform. 2026 Oct 20;6:1944540. doi: 10.3389/fbinf.2026.1944540 (PMC13728347; doi:10.3389/fbinf.2026.1944540)
Supplement: Supplementary file 1 [file Table2.docx]

Additional File: Table S2

| Category | Tool | Version | Reference |
| --- | --- | --- | --- |
| Alignment | BWA-MEM2 | v2.0 | [1] |
| Alignment | STAR | v2.7.11b | [2] |
| Processing | Picard tools | v1.129 | [3] |
| Processing | GATK | v4.3.0.0 | [4] |
| VCF Handling | BCFtools | v1.9 | [5] |
| VCF Handling | vt | v0.57721 | [6] |
| Quantification | featureCounts | v2.0.2 | [7] |
| Annotation | Ensembl VEP | 114 | [8] |
| Expression Outliers | OUTRIDER | 1.28.1 | [9] |
| Aberrant Splicing | FRASER | 2.6.1 | [10] |
| ASE Analysis | MBASED | 1.44.0 | [11] |
| Authentication | SNPRelate | 1.44.0 | [12] |
| Visualization | igvShiny | 1.6.0 | [13] |
| Data Handling | GenomicRanges | 1.62.1 | [14] |

1.	Vasimuddin, M., et al. *Efficient Architecture-Aware Acceleration of BWA-MEM for Multicore Systems*. in *2019 IEEE International Parallel and Distributed Processing Symposium (IPDPS)*. 2019.

2.	Dobin, A., et al., *STAR: ultrafast universal RNA-seq aligner.* Bioinformatics, 2013. **29**(1): p. 15-21.

3.	Broad Institute, *Picard toolkit*, in *Broad Institute, GitHub repository*. 2019.

4.	Auwera, G.v.d. and B.D. O'Connor, *Genomics in the cloud : using Docker, GATK, and WDL in Terra*. First edition. ed. 2020, Sebastopol, CA: O'Reilly Media.

5.	Danecek, P. and S.A. McCarthy, *BCFtools/csq: haplotype-aware variant consequences.* Bioinformatics, 2017. **33**(13): p. 2037-2039.

6.	Tan, A., G.R. Abecasis, and H.M. Kang, *Unified representation of genetic variants.* Bioinformatics, 2015. **31**(13): p. 2202-4.

7.	Liao, Y., G.K. Smyth, and W. Shi, *featureCounts: an efficient general purpose program for assigning sequence reads to genomic features.* Bioinformatics, 2014. **30**(7): p. 923-30.

8.	McLaren, W., et al., *The Ensembl Variant Effect Predictor.* Genome Biol, 2016. **17**(1): p. 122.

9.	Brechtmann, F., et al., *OUTRIDER: A Statistical Method for Detecting Aberrantly Expressed Genes in RNA Sequencing Data.* Am J Hum Genet, 2018. **103**(6): p. 907-917.

10.	Scheller, I.F., et al., *Improved detection of aberrant splicing with FRASER 2.0 and the intron Jaccard index.* Am J Hum Genet, 2023. **110**(12): p. 2056-2067.

11.	Mayba, O., et al., *MBASED: allele-specific expression detection in cancer tissues and cell lines.* Genome Biol, 2014. **15**(8): p. 405.

12.	Zheng, X., et al., *A high-performance computing toolset for relatedness and principal component analysis of SNP data.* Bioinformatics, 2012. **28**(24): p. 3326-8.

13.	Shannon, P.G., A.; Scigocka, K., *igvShiny: igvShiny: a wrapper of Integrative*

*Genomics Viewer (IGV - an interactive tool for visualization and exploration integrated*

*genomic data)*. 2025.

14.	Lawrence, M.H., W.; Pages, H.; Aboyoun, P.; Carlson, M., and e. al., *Software for Computing and Annotating Genomic Ranges*. 2013.
